# The η-secretase-derived APP fragment ηCTF is localized in Golgi, endosomes and extracellular vesicles and contributes to Aβ production

**DOI:** 10.1007/s00018-023-04737-4

**Published:** 2023-03-17

**Authors:** Elissa Afram, Inger Lauritzen, Alexandre Bourgeois, Wejdane El Manaa, Eric Duplan, Mounia Chami, Audrey Valverde, Bauer Charlotte, Raphaëlle Pardossi-Piquard, Frederic Checler

**Affiliations:** 1 Université Côte d’Azur, INSERM, CNRS, IPMC, UMR7275, Team Labeled “Laboratory of Excellence (Labex) DISTALZ”, 660 route des Lucioles, 06560 Sophia-Antipolis, Valbonne France; 2 Present Address: Fonds de Dotation CLINATEC, 17 rue des Martyrs, Bat 43, 38054 Grenoble, France

**Keywords:** Alzheimer’s disease, ηCTF, Endosomes, Extracellular vesicles, Autophagic degradation, Aβ-production

## Abstract

The processing of the amyloid precursor protein (APP) is one of the key events contributing to Alzheimer’s disease (AD) etiology. Canonical cleavages by β- and γ-secretases lead to Aβ production which accumulate in amyloid plaques. Recently, the matrix metalloprotease MT5-MMP, referred to as η-secretase, has been identified as a novel APP cleaving enzyme producing a transmembrane fragment, ηCTF that undergoes subsequent cleavages by α- and β-secretases yielding the Aηα and Aηβ peptides, respectively. The functions and contributions of ηCTF and its related fragments to AD pathology are poorly understood. In this study, we designed a novel immunological probe referred to as ηCTF-NTer antibody that specifically interacts with the N-terminal part of ηCTF targeting ηCTF, Aηα, Aηβ but not C99, C83 and Aβ. We examined the fate and localization of ηCTF fragment in various cell models and in mice. We found that overexpressed ηCTF undergoes degradation in the proteasomal and autophagic pathways and accumulates mainly in the Golgi and in endosomes. Moreover, we observed the presence of ηCTF in small extracellular vesicles purified from neuroblastoma cells or from mouse brains expressing ηCTF. Importantly, the expression of ηCTF in fibroblasts devoid on APP leads to Aβ production demonstrating its contribution to the amyloidogenic pathway. Finally, we observed an ηCTF-like immunoreactivity around amyloid plaques and an age-dependent accumulation of ηCTF in the triple-transgenic mouse AD model. Thus, our study suggests that the ηCTF fragment likely contributes to AD pathology by its exosomal spreading and involvement in Aβ production.

**Supplementary Information:**

The online version contains supplementary material available at 10.1007/s00018-023-04737-4.

## Introduction

Among all hypotheses proposed to explain the etiology of Alzheimer’s disease (AD), the amyloid hypothesis represents the most widely accepted one since it is supported by a large number of biochemical and histopathological evidences. This amyloid cascade hypothesis postulates that accumulation and aggregation of hydrophobic amyloid β peptides (Aβ) trigger synaptic dysfunction and neurodegeneration in Alzheimer’s disease [1, 2]. Genetic evidences also provided strong support to this amyloid cascade. Thus, most of APP mutations responsible for aggressive autosomal dominant familial forms of AD (FAD) lead to exacerbated production of Aβ or yield aggregation-prone Aβ species [3]. Conversely the “Icelandic” APP mutation (APP Ala673Thr) decreases Aβ levels by about 40% and protects against the risk of developing AD [4].

How APP is proteolytically processed has been the matter of numerous studies. APP undergoes a complex set of proteolytic events by enzymatic activities called secretases. Two main proteolytic pathways referred to as amyloidogenic and non-amyloidogenic pathways have been extensively examined. In the amyloidogenic pathway, APP is first cleaved by the β-secretase to release a C-terminal fragment, C99, which then undergoes subsequent hydrolysis by the γ-secretase to yield not only Aβ but also the cytosolic APP Intracellular Domain (AICD) that controls the transcription of several genes [5]. However, this amyloidogenic pathway responsible for Aβ production does not represent the major part of APP metabolism. The non-amyloidogenic pathway that precludes Aβ formation is the major APP processing route. It involves α-secretase, yielding a C83 fragment that is further cleaved by γ-secretase, thereby producing AICD and a small N-terminally truncated fragment named p3.

In addition to these two canonical and well-described pathways, a newly η-secretase pathway has emerged. First Wang and collaborators reported in HEK293 cells an accumulation of two novel APP-CTF fragments at 15 and 25 kDa unraveled upon cathepsins inhibition [6]. They were the first to name the 25 kDa APP-CTF fragment as the new APP-CTFη or ηCTF fragment [6]. Using various antibodies and mass spectroscopy analyses, Willem and colleagues subsequently demonstrated that this ηCTF fragment can be cleaved by α- and β-secretases to generate Aηα and Aηβ peptides, respectively [7]. They proposed the membrane-type matrix metalloprotease MT5-MMP as a new η-secretase since they observed a reduction of Aηα production in brains from MT5-MMP-knockout mice [7]. By distinct approaches, concomitant studies also demonstrated the cleavage of APP by MT5-MMP and showed its contribution to AD pathology. Thus, MT5-MMP deficiency reduced amyloid pathology, neuroinflammation and cognitive decline in 5xFAD mice [8, 9]. However, few data were available concerning the fate, biology and cellular localization of these η-secretase derived fragments.

In this current study, we observed that overexpressed ηCTF undergoes proteasomal and autophagic degradations in human neuroblastoma cells (SH-SY5Y) and for the first time, we show that ηCTF behaves as a genuine precursor of Aβ. Since, the immunological toolbox available to specifically detect ηCTF, particularly in in situ approach fell short, we designed a novel immunological probe referred to as ηCTF-NTer antibody that interacts selectively with ηCTF N-terminus thus targeting ηCTF, Aηα, Aηβ but importantly, not C83, C99 or AICD. Using this antibody in Hela transfected cells, we were able to establish a subcellular localization of ηCTF mainly in trans-Golgi network but also, to a smaller extent, in endosomes. This endosomal localization was also observed in organotypic hippocampal slices infected with an adeno-associated-virus (AAV) expressing ηCTF. In agreement, we detected the presence of ηCTF in small extracellular vesicles (sEVs) prepared from culture media of SH-SY5Y expressing APP or from brain tissues of AAV-ηCTF-injected mice. Of interest, microscopic analysis of ηCTF-NTer-like immunoreactivity revealed an ηCTF staining surrounding amyloid plaques associated to an age-dependent accumulation of ηCTF in cortex and hippocampus of triple transgenic mice (3xTgAD).

## Materials and methods

### Design of plasmid constructs and viruses:

#### pcDNA3 plasmid expressing Aηα or Aηβ

Aηα and Aηβ constructs were obtained by PCR amplification using APP695 cDNA as template and the forward primer 5ʹ-GATAAGCTTGCCACCATGATTAGTGAACCAAGGATCAGTTAC-3ʹ for both Aηα and Aηβ. This primer contains a Hind III restriction site and a Kozak sequence. We used the reverse primer 5ʹ-GATCTCGAGCTATTTTTGATGATGAACTTCATATCCTGAGTC-3ʹ for Aηα and the reverse primer 5ʹ-GATCTCGAGCTACATCTTCACTTCAGAGATCTCCTCC-3ʹ for Aηβ. Both reverse primers contain the XhoI enzyme restriction site. The amplicon was then digested by Hind III and XhoI enzymes and subcloned in the pcDNA3 vector.

#### pcDNA3 plasmid expressing ηCTF 

the ηCTF plasmid construction contains the APP signal sequence and two additional residues (Leu-Glu) from APP695 cDNA in frame with the 5ʹ end of the ηCTF sequence. As previously described for SPA4CT [10], SP-C99 and SP-C83 constructs [11], the APP signal sequence is required for a correct membrane insertion of the ηCTF fragment. In a first PCR reaction, the APP signal sequence was amplified using the primers 5ʹ-GATAAGCTTATGCTGCCCGGTTTGGCACT-3ʹ that contains an HindIII restriction site and 5ʹ-TTCACTAATCATCTCCAGCGCCCGAGCC-3ʹ containing the first nucleotides of the 5’ end of ηCTF sequence. In an additional PCR, ηCTF sequence was amplified using the primer 5ʹ-GCGCTGGAGATGATTAGTGAACCAAGGATCAGTTA-3ʹ that contains the last nucleotides of the 3ʹ end of the signal peptide sequence, and the primer 5ʹ-GATCTCGAGCTAGTTCTGCATCTGCTCAAAGAA-3ʹ containing XhoI restriction site. Finally, the amplicons resulting from the first two PCR were incubated together in a third PCR reaction to obtain a final DNA fragment containing the APP signal peptide in frame with ηCTF. This amplicon digested with HindIII and XhoI enzyme was then subcloned in pcDNA3 vector.

#### AAV-10 plasmid expressing ηCTF under the control of synapsin-1 promoter

For viral plasmid construction, the pcDNA3 plasmid expressing ηCTF described above was used as a template with the primers 5ʹ-GATGCTAGCCCACCATGCTGCCCGGTTTGGCACTGCTCCT-3ʹ and 5ʹ-GATGCTAGCCTAGTTCTGCATCTGCTCAAAGAACTTGTAGGTT-3ʹ that both contain a NheI restriction enzyme site. The PCR product was then digested with NheI and sub-cloned into AAV10 plasmid.

#### pGEX plasmid for expression of recombinant ηCTF

the DNA sequence encoding the ηCTF was amplified by PCR, using the forward primer 5ʹ-GATGGATCCATGATTAGTGAACCAAGGATCAGTTA-3ʹ, which has a BamH1 restriction site and the reverse primer 5ʹ-GATCTCGAGCTAGTTCTGCATCTGCTCAAAGAA-3ʹ, containing a XhoI restriction site. The PCR product was then sub-cloned between BamH1 and XhoI restriction enzymes sites of pGEX-4T-1 vector.

All above constructs were verified by full sequencing.

### Recombinant ηCTF production

Recombinant ηCTF fragment was obtained as previously described for the production of recombinant PrP^c^ fragment N1 [12], with some minor changes. Briefly, pGEX-4T-1 vector containing ηCTF sequence was transformed into BL21 gold strain of Escherichia coli. After induction with Isopropyl 1-thio-d-galactopyranoside (200 μM), the medium was centrifuged. Cells pellets were resuspended with PBS supplemented with a protease inhibitor mixture, DTT (10 mM) and lysozyme (0.2 mg/ml), then proteins were solubilized by the addition of Triton X-100 (10%), MgCl2 (1 M), and DNase (1u/μl). After centrifugation, glutathione-sepharose beads (GE Healthcare) were added to the supernatant, pelleted, and resuspended in PBS (1 ml). Peptides were cleaved with thrombin (5 units/ml; GE Healthcare), and thrombin was removed using Sepharose benzamidine beads (GE Healthcare).

### Production of the ηCTF-Nter antibody

The new ηCTF-Nter antibody is a rabbit polyclonal antibody made following the Covalab’s immunization protocol. As immunogen epitope, we designed peptide corresponding to the first 16 N-terminal residues of ηCTF fragment, the C-terminal end of which was blocked by a cystein residue to preserve a free N-terminal part of the peptide and to obtain antibodies directed towards the N-terminal moiety (sequence: MISEPRISYGNDALM-C). Rabbit’s immunoreactivity and titer were controlled by ELISA then the ηCTF-Nter antibody was purified by antigen-specific affinity with the same peptide used for immunization.

### Viral production and mice ICV injection:

Virus production was performed following a protocol previously described [13]. Briefly, HEK293 cells were transfected with the adenovirus helper plasmid (pXX6), the AAV packaging plasmid (rAAV2-rh10), and the AAV10 plasmid empty vector or encoding human ηCTF under control of the synapsin-1 promoter (AAV-empty, and AAV- ηCTF). Viruses were produced, purified and vector titers were determined by real-time PCR and expressed as viral genomes per ml (vg/ml). Four µl of AAV virus (5.5 × 10^12^vg/ml) were administrated in 1-day-old C57Bl6JRj mice (Janvier Labs, France) through intra-cerebroventricular (ICV) injection as described previously [13] then mice brains were analyzed at 3 months of age by western blot and immunochemistry.

### Animals

Pregnant C57Bl6JRj females (Janvier Labs, France) were ordered for new born mice viral injection (see above). In addition wild-type and 3xTg (APP_swe_; Tau_P301L_; PS1_M146V_) mice were maintained from breeding pairs provided by Dr F. LaFerla [14]. All mice were kept on the original 129/C57Bl6 background strain, backcrossed every 10 generations and genotyped. Animals were housed with 12 h/12 h light/dark cycle and were given free access to food and water. All experimental procedures were in accordance with the European Communities Council Directive of 22 September 2010 (2010/63/EU) and approved by the French Ministry of Higher Education and Research (project number APAFIS#9766-201704261624789.v3).

### Cell Culture and treatments

Human neuroblastoma (SH-SY5Y, ATTC), human epitheloid cervix carcinoma (HeLa, ATCC) and mouse embryonic fibroblasts either wild type (MEF-APPwt), or naturally devoid of APLP1 and knocked out for APP and APLP2 [15] (referred to as MEF APP KO) were cultured in Dulbecco’s modified Eagle’s medium supplemented with fetal calf serum (10%), penicillin (100 U/ml) and streptomycin (50 μg/ml), and incubated at 37 °C in a 5% CO_2_ atmosphere. Human SH-SY5Y cells stably expressing wild-type full-length APP (SH-SY5Y-APPWT) were generated as already described [16], and maintained in the presence of G-418 (400 µg/ml). Transient transfections of cells were carried out using Lipofectamine 2000 (Life Technologies) for SH-SY5Y and MEFs and JetPrime (Polyplus transfection) for HeLa cells, according to the manufacturer’s instructions. Twenty-four hours post-transfection, cells were treated with pharmacological agents: Lactacystin (5 µM; Sigma-Aldrich), Epoxomicin (1 µM; Enzo), MG132 (5 µM), Bafilomycin A1 (100 nM), Smer28 (50 µM), or with secretases inhibitors: β-secretase inhibitor, Bi (30 µM, Elan Pharmaceuticals), α-secretase inhibitor, Gi (GI254023X; 10 µM; Sigma-Aldrich) or γ-secretase inhibitor D6 (1 µM; Elan Pharm/Imago). Cells were analyzed 48 h post-transfection.

### Western-blot analysis

Cells were lysed in RIPA buffer [Tris (50 mM); pH 7,4; NaCl (150 mM); EDTA (1 mM); Triton X100 (1%); deoxycholate (0.5%); SDS (0.1%)] supplemented with a cocktail of protease inhibitors (Roche diagnostics). Cell homogenate proteins (50 µg) were separated on 16% tris-tricine gels, and sEVs proteins were separated on 16% tris-tricine gels or Bio-Rad 12% stain-free TM TGX FastCastTM acrylamide gels. It should be noted that in few cases, when protein of interests and loading control proteins have similar molecular weights, to achieve accurate normalizations, gels were run separately in strictly identical conditions. All full gels are provided as indicated in the legends of figures showing gels. Tris-tricine gels were directly transferred to nitrocellulose membranes using a conventional transfer system and boiled in PBS before saturation. Bio-Rad gels were photoactivated for the visualization of proteins before being electrophoretically transferred to nitrocellulose membranes using the Bio-Rad Trans-Blot^®^ TurboTM Transfer System. Membranes were saturated with skimmed milk, and incubated overnight with the following primary antibodies: APPCter rabbit polyclonal (1:5000, gift from Paul Fraser); WO2 mouse monoclonal (1:5000, Sigma–Aldrich); ηCTF-Nter rabbit polyconal (1:1000, homemade with Covalab, see above); α-HSC70 mouse monoclonal (1:1000, Santa Cruz) or α-GAPDH mouse monoclonal (1:5000, EMD Millipore). After probing with primary antibodies, immunological complexes were revealed with anti-mouse or anti-rabbit HRP-conjugated antibodies (1:5000, Jackson ImmunoResearch) followed by electrochemiluminescence (Westernbright™ Sirius™ and Quantum™ chemiluminescent HRP substrate, Advansta, France). Peak height of signal intensities from protein bands were quantified with MultiGauge software.

For western-blot analysis of mice brains, following intracardiac perfusion with PBS, hemibrains were extracted then enzymatically and mechanically lysed using “the adult brain dissociation kit” and a GentleMACS Dissociator (Miltenyi Biotec). PBS was added to the cell suspension, which was then filtered through a 70 μm Smartstrainer and centrifuged at 300×*g* for 10 min. The cell pellets were lysed in RIPA buffer supplemented with protease inhibitors and then loaded on Bio-Rad 12% stain-freeTM TGX FastCastTM acrylamide gels.

### Sandwich ELISA analysis

MEF APP KO cells were transiently transfected with pcDNA.3 or ηCTF constructs using Lipofectamine 2000 and treated or not with secretases inhibitors Bi, Gi and D6. The concentrations of human secreted Aβ40 were measured in the culture medium using the ELISA kit (Invitrogen) following the manufacturer’s instructions.

### Aηα immunoprecipitation

MEF APP KO cells were plated on 6 wells plates, transiently transfected with pcDNA.3 or ηCTF constructs using Lipofectamine 2000 and treated or not with secretases inhibitors Bi, Gi and D6, as described above. Culture media were collected, centrifuged at 14,000×*g* for 5 min to remove cell debris then supernatants were supplemented with RIPA buffer 5X and precleared with 10 μl of Protein A Sepharose (GE Healthcare) for 1 h at 4 °C. Resulting supernatants were incubated for 1 h with WO2 mouse monoclonal antibody (1 μl) then 20 μl of Protein A Sepharose beads were added and left for overnight incubation. After three washes with RIPA buffer (and 1 time in PBS), the beads were resuspended with 20 μl of loading buffer, loaded on a tris-tricine gel (16%) and subjected to western-blot analysis as described above.

### Cells immunostaining

Hela cells grown on coverslips were fixed in paraformaldehyde 4% solution for 10 min, permeabilized with Triton-X 100 (0.1%) for 10 min, saturated in BSA (5%)/Tween20 (0.1%), and probed for 1 h with appropriate primary antibodies: APPCter rabbit polyclonal (1:5000), APPCter, mouse monoclonal (1:5000, Biolegend), WO2 mouse monoclonal (1:5000), or ηCTF-Nter rabbit polyclonal (1:1000) for ηCTF detection, α-Lamp2 mouse monoclonal (1:1000, Santa Cruz), α-EEA1 rabbit monoclonal (1:200, Cell Signaling Technology), α-CD63 mouse monoclonal (1:1000, Santa Cruz) and α-TGN46 sheep polyclonal (Serotec, AHP500G, 1:1000) for lysosomes, early and late endosomes and golgi specific markers, respectively. After washes, coverslips were incubated for 1 h with Alexa Fluor-488 and Alexa Fluor-590 conjugated antibodies (Molecular Probes, 1:1000) and DAPI (1:20,000, Roche) staining. Finally, the sections were washed with PBS, then mounted onto glass slides and cover-slipped. The stained slices were kept at 4◦C before analysis with confocal microscopy (Zeiss LSM 780 with 63 × Objective).

### DAB and immunofluorescence staining of tissue

For immunohistochemistry, mice were anesthetized by an intraperitoneal injection of a combination of ketamine (150 mg/kg), xylazine (10 mg/kg) and acepromezine (10 mg/kg) then perfused intra-cardiacally with PBS followed by paraformaldehyde (4%) before collecting the brains. Paraformaldehyde-fixed brains were embedded in paraffin and cut on a microtome in 8 μm thick sections (Thermoscientific, France). Brain sections were deparaffined in xylen bath and rehydrated by successive 5 min baths of EtOH (100%, twice), 90%, and then 70%. Antigens were unmasked in a 90% formic acid bath for 5 min and then in citric acid solution at 90–100 °C for 20 min (Pressure cooker, Vector Laboratories). For DAB staining, sections were treated with H_2_O_2_ 3% for 15 min. Non-specific binding sites were blocked for 1 h in BSA (5%)/Tween20 (0.1%) and brain sections were then incubated at 4 °C overnight with primary antibodies ηCTF-Nter rabbit polyclonal (1: 800) or WO2 mouse monoclonal (1:1000). Sections were incubated for 1 h with Alexa Fluor-488 and Alexa Fluor-594 conjugated antibodies (1:1000) and DAPI (1:20,000) for immunofluorescence, or with horseradish peroxidase (HRP)-conjugated antibodies (Jackson ImmunoResearch, 1:1000) then revealed with the DAB-ImmPACT system (Vector Laboratories) for DAB-staining. Images were taken with a confocal Leica TCS SP5 microscope (Immunofluorescence) or with DMD108 Leica microsystem (DAB) and processed using ImageJ software. For ηCTF-Nter staining, signal was amplified with Avidin Biotin Complex (ABC) method (VECTASTAIN Original ABC Kit, Vector Laboratories).

### Purification of exosomes (sEVs)

SH-APPWT cells were cultivated in DMEM for 24 h, rinsed twice with PBS, and treated or not with the β-secretase inhibitor Bi or Bafilomycin A1 as described above, for another 24 h in OptiMEM to allow vesicle secretion. When overexpressing ηCTF, cells were transiently transfected 1 day before secretion in OptiMEM. sEVs were purified by ultracentrifugation as previously described [17]. Briefly, media were first harvested and centrifuged at 2000×*g* for 20 min, then filtered through a 0.22 μm filter. The supernatant was then sequentially centrifuged at 10,000×*g* for 30 min and 100,000×*g* for 125 min. The obtained pellet containing sEVs and contaminating proteins was washed in ice-cold PBS and centrifuged for another 120 min. The final sEVs pellet was resuspended in RIPA buffer and sonicated in an ultrasonic bath for 15 min before western-blot analysis. sEVs isolated from the extracellular space of AAV-ηCTF mice brain were purified according to the protocol previously described [17]. Briefly, intracardiac perfusion with PBS was performed then hemibrains were extracted and enzymatically and mechanically lysed using “the adult brain dissociation kit” and a GentleMACS Dissociator (Miltenyi Biotec). PBS was added to the homogenate suspension, which was then filtered through a 70 μm Smartstrainer and centrifuged at 300×*g* for 10 min. The supernatant was used for sEVs purification and the cell pellet was kept for further analysis by western blot as described above. For sEVs purification, the supernatant was sequentially centrifuged several times as previously described [16], and the last pellet was loaded on a sucrose gradient and centrifuged at 200,000×*g* for 16 h. The fractions containing the sEVs were collected, diluted with PBS and centrifuged at 100,000×*g* for 90 min. The final sEVs pellet was resuspended in PBS, lysed in RIPA buffer complemented with protease inhibitors and ultrasonicated before western-blot analysis. Before loading, nanoparticle analysis was performed on each fraction using the ZetaView instrument (Particle-Metrix) to determine particle size distribution and concentration.

### Membrane fraction preparation

Dissected hippocampi of 3xTgAD (AD) and wild-type (WT) mice were homogenized in hypotonic buffer [HEPES (5 mM), pH 7.4, EDTA (1 mM), sucrose (0.25 M) containing a proteases inhibitor cocktail]. The homogenate was centrifuged at 1000×*g* for 5 min at 4 °C and the supernatant was centrifuged at 100,000×*g* for 1 h. Membrane pellets were then solubilized in RIPA buffer complemented with protease inhibitor, and centrifuged at 100,000×*g* for 20 min. Supernatants were recovered as the soluble membrane fractions and loaded on 16% tris-tricine gels.

### Statistical analyses

Statistical analyses were performed using Prism software (GraphPad Prism 7). Quantitative data are represented as means ± SEM and subjected to non-parametric tests such as the Mann–Whitney test for single comparisons and the Tukey one-way ANOVA test for multiple comparisons. Statistical significance code is: ****p < 0.0001 and *p < 0.05.

## Results

### Proteasomal and autophagic degradation of ηCTF in human SH-SY5Y neuroblastoma cells

**Fig. 1:**
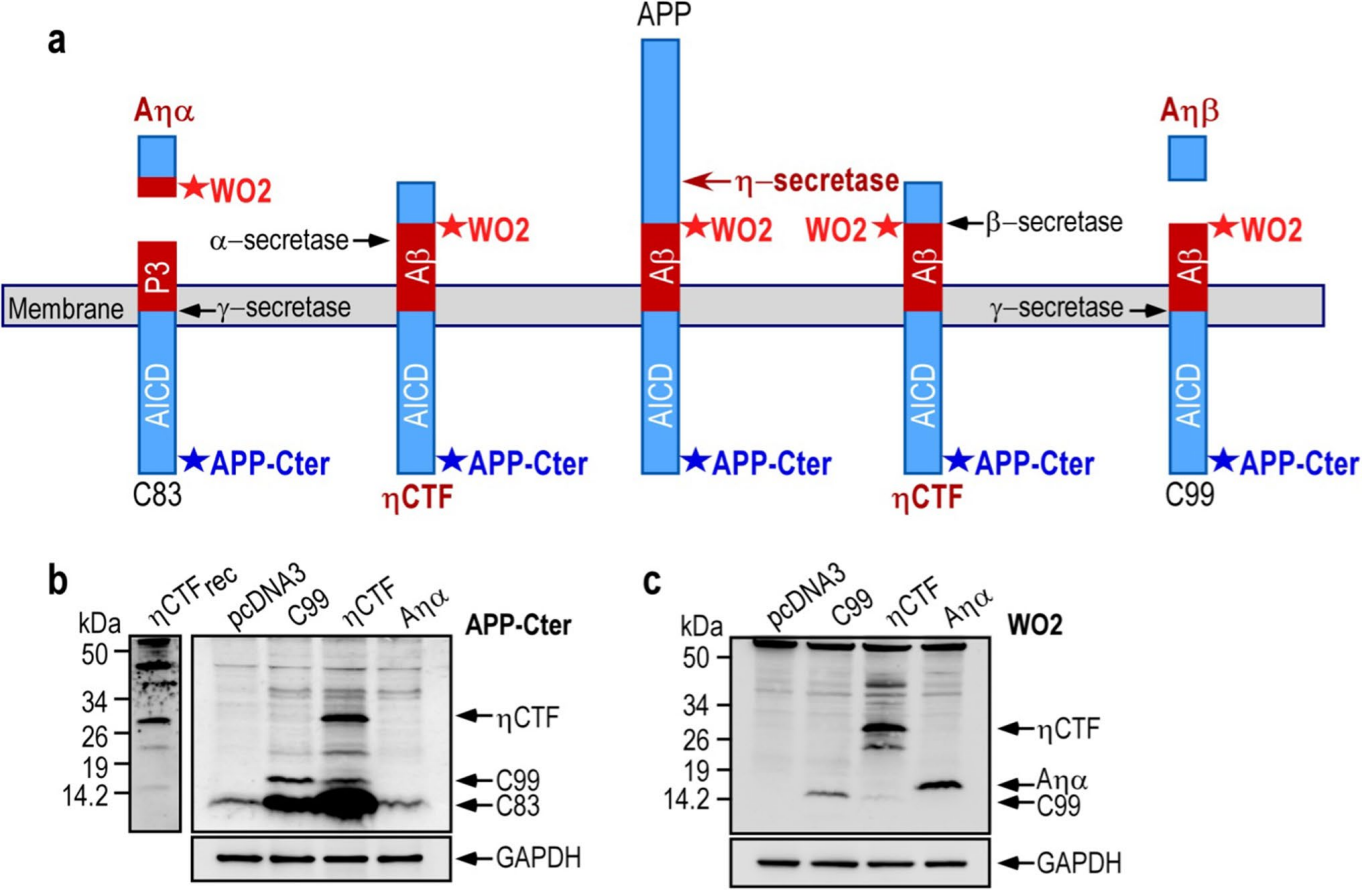
Expression and detection of ηCTF in SH-SY5Y cells.** a** Schematic illustration of APP-derived η-secretase-mediated production of ηCTF and its subsequent cleavages by indicated secretases. Stars indicate the sites of recognition of proteolytic fragments or peptides by WO2 and APP-Cter antibodies. The η-secretase cleaves APP protein to produce a transmembrane fragment called ηCTF that undergoes subsequent cleavages by β and/or α-secretases yielding Aηβ and Aηα respectively. The WO2 antibody targets the amino acid residues 4–10 of Aβ peptide and recognizes full-length APP, ηCTF, C99, Aηα but neither C83 nor Aηβ. The APP-Cter antibody is directed toward the last residues of APP protein and recognizes full-length APP, ηCTF, C99, C83 but neither Aηα nor Aηβ. **b**–**c** SH-SY5Y cells were transiently transfected with C99-, ηCTF-, Aηα-bearing vectors or empty pcDNA3 vector and analyzed by western blot using APP-Cter (**b**) or WO2 (**c**) antibodies. A specific band corresponding to ηCTF is detected around 30 kDa by both APP-Cter and WO2 antibodies. Recombinant ηCTF protein (ηCTFrec) is used as molecular weight control and GAPDH as loading control. All full gels are provided in Sup Fig. 5

A newly described η-secretase cleavage occurs on APP_695_ between residues 504 and 505, yielding a 95 kDa soluble fragment (sAPPη or sAPP95) and a 25–30 kDa transmembrane fragment called ηCTF, which is subsequently cleaved by α- or β-secretases to generate Aηα and Aηβ peptides, respectively (Fig. 1a). In to understand the biology and fate of the ηCTF fragment, we expressed it in human neuroblastoma cells (SH-SY5Y) and analyzed its expression by western blot using APP-Cter and the WO2 antibodies targeting, the carboxy terminal end of APP and the amino-terminal part of Aβ peptides, respectively (Fig. 1a). In SH-SY5Y cells transiently transfected with the ηCTF-pcDNA coding vector, a specific band around 30 kDa is detected by both APP-Cter and WO2 that displays a similar migrating pathway than recombinant ηCTF (Fig. 1b, c), thus firmly supporting the identity of ηCTF. As expected, in C99-transfected SH-SY5Y cells, C99 is also recognized by these two antibodies (Fig. 1b, c) while APP-Cter antibody, but not WO2, detects endogenous C83 (Fig. 1b). In both C99 and ηCTF overexpressing cells, the C83 levels are increased as compared to control cells, indicating that both of these fragments undergo an α-secretase-mediated cleavage (Fig. 1b). Similarly, in ηCTF overexpressing cells, the C99 levels are increased as compared to control cells indicating that ηCTF also behaved as a β-secretase substrate (Fig. 1b). Of note, as expected, WO2 but not APP-Cter recognizes the Aηα peptide (Fig. 1c). This set of experiments that aimed at characterizing ηCTF expression in SH-SY5Y cells indicates that ηCTF mostly undergoes α- and β-secretase cleavages giving rise to C99 and C83 as previously described [7] while the ηCTF-derived production of Aηα remains poorly detectable in cell homogenates.

**Fig. 2:**
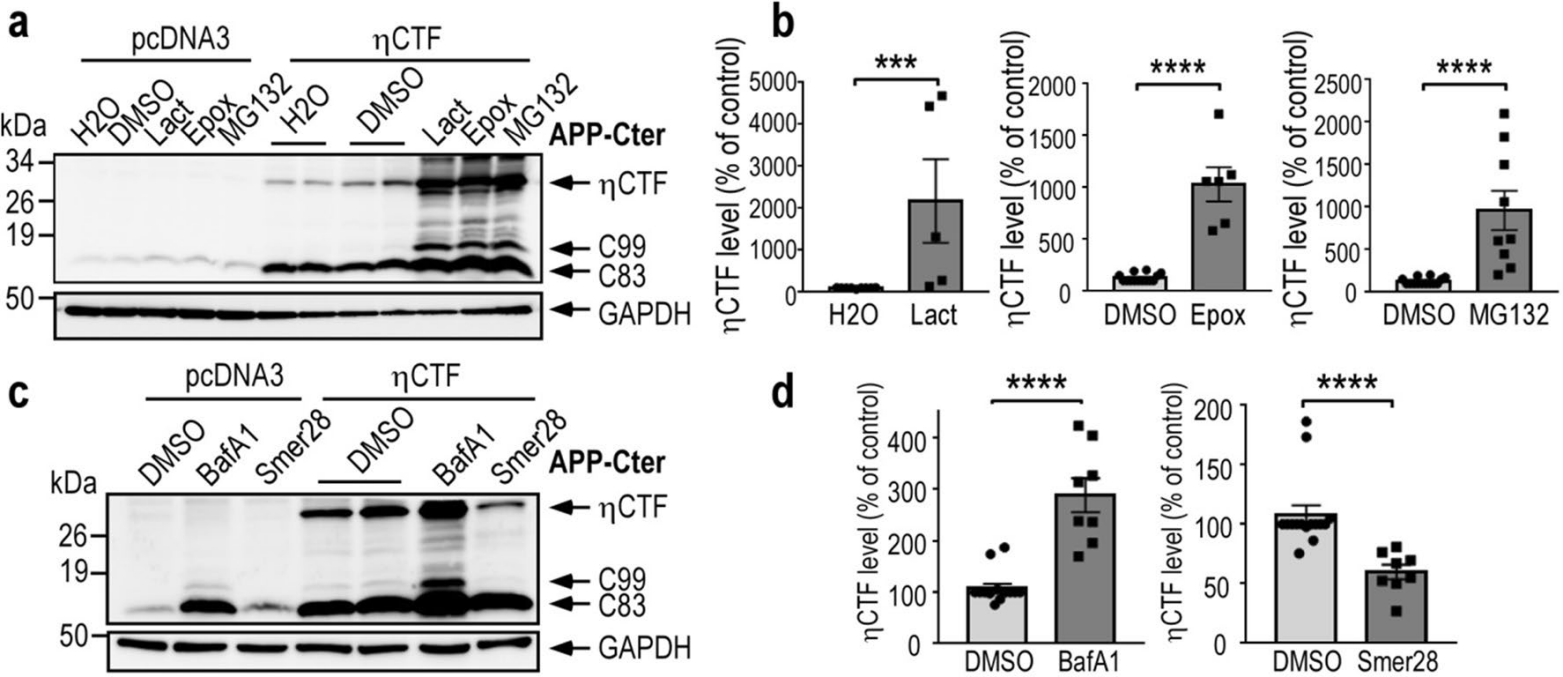
ηCTF fragment is degraded by both proteasome and autophagic pathways. **a**–**d** SH-SY5Y cells were transiently transfected with ηCTF or pcDNA3 vectors and treated for 24 h with proteasome inhibitors (**a**, **b**, lactacystine (Lact, 5 µM), epoxomicin (Epox, 1 µM), MG132 (5 µM)) or with bafilomycin A1 (BafA1, 100 nM) or Smer28 (50 µM) that blocks or activates autophagy respectively (**c**, **d**) then analyzed by western blot using APP-Cter antibody. Histograms in **b**, **d** correspond to the quantification of ηCTF immunoreactivity obtained in **a**,** c** and are expressed as percent of controls (H_2_O or DMSO-treated cells) taken as 100. Bars are the means ± SEM of 5–9 independent determinations. ****p < 0.0001 according to Mann–Whitney test. All full gels are provided in Sup Fig. 5

To uncover the cellular pathways by which ηCTF is degraded, we used a pharmacological approach and treated ηCTF expressing SH-SY5Y cells with several well characterized inhibitors targeting either proteasomal or autophagic degradative processes. All proteasome inhibitors namely, lactacystin, epoxomicin or MG132 lead to significant increases of ηCTF expression (Fig. 2a, b), thus demonstrating the involvement of the proteasomal machinery in ηCTF degradation in SH-SY5Y cells. In addition, two lines of evidences indicated significant additional contribution of the autophagic pathway. First, ηCTF immunoreactivity is increased by the potent and selective autophagy blocker bafilomycin A1 (Fig. 2c, d). Second, ηCTF expression is decreased by the activator of autophagy SMER28 (Fig. 2c, d). Inhibition of both proteasome and autophagy also leads to an accumulation of endogenous and ηCTF-derived C99 and C83 fragments (Fig. 2a, c), which have already been described to be degraded by these two protein degradation systems [13, 18–21]. Overall, both proteasomal and autophagic pathways contribute to ηCTF degradation overexpressed in human neuronal cells. However, since the increase in endogenous C83 expression is more importantly obtained by bafilomycin A1 treatment (Fig. 2c), this suggests that the autophagic process likely remains the main degradative process of endogenous APP-CTF. Accordingly, C83, C99 and a band corresponding to ηCTF are also increased following bafilomycin A1 treatment of SH-SY5Y cells stably expressing wild-type APP protein (SH-APPWT, Sup Fig. 1a).

We aimed at characterizing the influence of secretases inhibitors on ηCTF processing but since in native SH-SY5Y cells, it is difficult to detect endogenous ηCTF immunoreactivity even after blockade of the proteasomal and autophagic pathways (Fig. 2a, c), we used SH-APPWT cells and treated them with α-, β- or γ-secretase inhibitors. We analyzed all APP-Cter fragments produced using APP-Cter, WO2 and 82E1 antibodies, the latter targeting specifically the free N-terminal of C99, and thus, is unable to detect ηCTF. As expected, the levels of C83 and C99 are reduced upon α- and β-secretase inhibitions by Gi and Bi, respectively, while both C83 and C99 immunoreactivities were increased when γ-secretase is blocked by its selective and potent inhibitor D6 (Sup Fig. 1b). Interestingly, a band migrating around 30 kDa detected by WO2 but not 82E1 antibody is noticeably increased following β-secretase inhibition in pcDNA_3_-transfected cells (Sup Fig. 1b, pcDNA3 lane Bi, see long exposures) while, as expected, β-secretase inhibition fully abolishes APPWT-derived C99 formation detected by both antibodies. This 30 kDa band increased by Bi and bafilomycin A1 treatments in pcDNA_3_ mock-transfected cells appears with a similar migrating pathway than specific ηCTF-like immunoreactivity obtained in ηCTF-expressing SH-APPWT cells and thus might correspond to the ηCTF fragment derived from the stably expressed wild-type APP protein (Sup Fig. 1a, b). Of note, none of the secretase inhibitors significantly affected the level of ηCTF in ηCTF-transfected SH-APPWT-cells (Sup Fig. 1b, c).

### Expression of ηCTF in fibroblasts devoid of APP leads to Aηα and Aβ productions

**Fig. 3:**
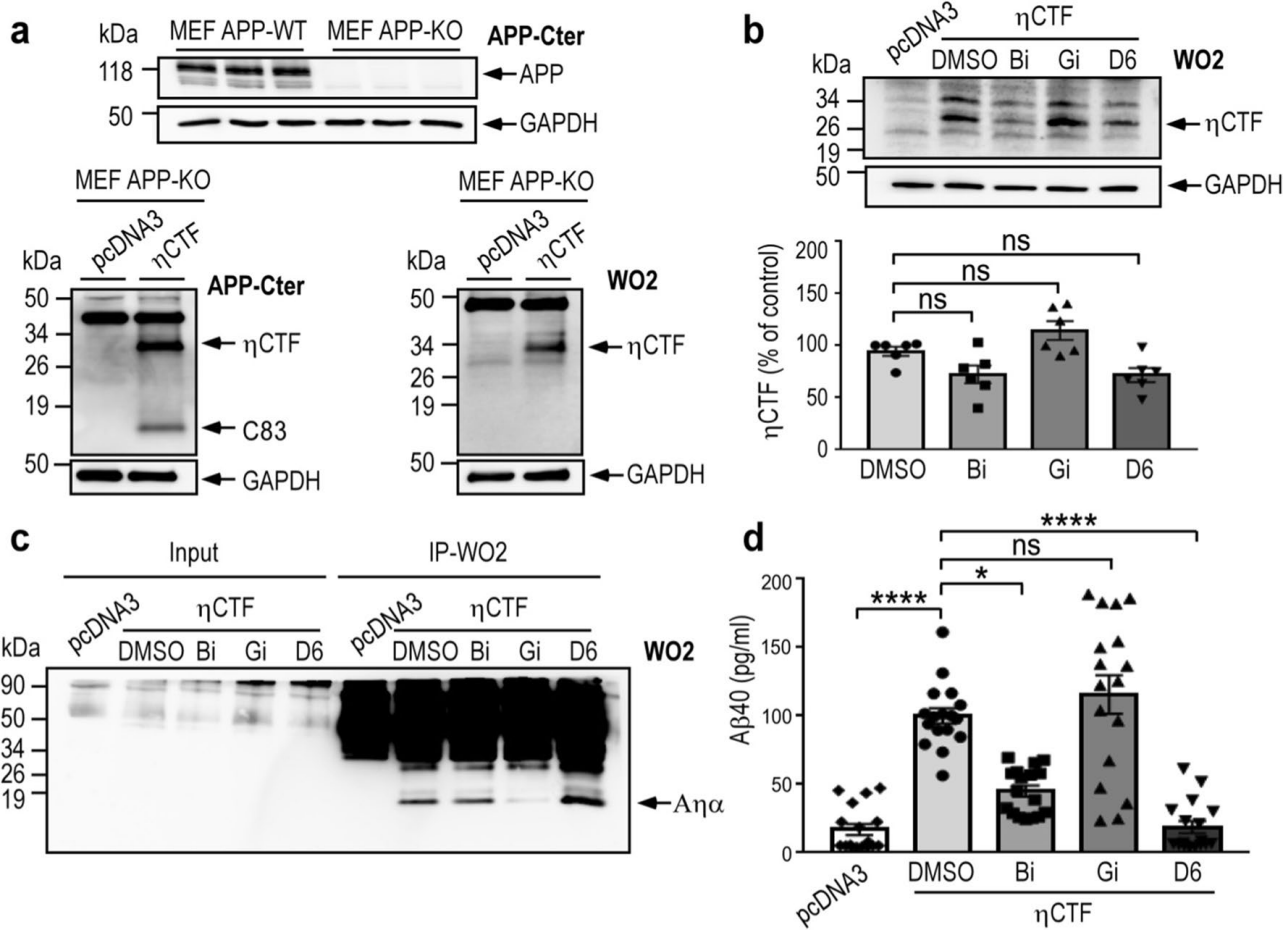
ηCTF fragment yields both Aηα and Aβ peptides. **a** Wild-type (MEF APPwt) and APP/APLPs-deficient mouse embryonic fibroblasts (MEF APPKO) were transiently transfected with ηCTF or pcDNA3 vectors and analyzed by western blot using APP-Cter and WO2 antibodies. GAPDH is used as loading control. **b**–**d** ηCTF transfected MEF APPKO cells were treated for 24 h with α- β- or γ-secretase inhibitors (Gi:10 µM, Bi:30 µM, D6:1 µM) then analyzed by western blot using WO2 antibody (**b**). GAPDH is used as loading control. Bars correspond to the quantification of ηCTF immunoreactivity expressed as percent of controls (DMSO-treated cells) taken as 100 and are the means ± SEM of 6 independent determinations. Ns, not statistically significant according to the Tukey one-way ANOVA test (**b**). Aηα peptides were immunoprecipitated (IP) using WO2 antibody from conditioned medium of MEF APP KO cells expressing or not ηCTF and treated with α- β- or γ-secretase inhibitors. Note that Aηα was not detectable in secretates before immunoprecipitation (Input) (**c**). Aβ40 levels were measured by ELISA in the conditioned medium of MEF APPKO cells expressing or not ηCTF and treated with α- β- or γ-secretase inhibitors. Bars indicate the concentration of Aβ in pg/ml and are the means ± SEM of 17 independent determinations. ****p < 0.0001, *p < 0.05, ns: not statistically significant according to the Tukey one-way ANOVA test (**d**). All full gels are provided in Sup Fig. 5

APP-CTF fragments (C99 and C83) may theoretically derive from both APP-full length or ηCTF in SH-SY5Y cells (see above). To delineate those genuinely derived from ηCTF, we expressed this fragment in mouse embryonic fibroblasts devoid of endogenous APP and its family members APLP1 and APLP2 (MEF APP KO, Fig. 3a). As expected, ηCTF is readily expressed in fibroblasts and detected by both the APP-Cter and the WO2 antibodies (Fig. 3a). We treated the ηCTF expressing APP KO fibroblasts with secretase inhibitors and examined the Aηα and Aβ productions. Although α-, β- or γ-secretase inhibitions do not significantly modulate ηCTF expression in MEF APP KO (Fig. 3b), we provide evidence that ηCTF is indeed processed by α- β- and γ-secretase activities. First, ηCTF expression in MEF APP KO leads to the production of an APP-CTer- but not WO2-immunoreactive fragment corresponding to C83 (compare lower panels in Fig. 3a). Second, we specifically immunoprecipitate a secreted fragment labeled by WO2 which is fully sensitive to the α-secretase inhibitor Gi, thus corresponding to Aηα (Fig. 3c). Third, for the first time, we were able to measure by ELISA an increased level of Aβ40 in ηCTF expressing APP KO fibroblasts compared to pcDNA3 control fibroblasts, and as expected, we observed a drastic decrease of Aβ40 upon cells treatment with either β- or γ-secretase inhibitiors (Fig. 3d). Although Aβ production indicates that ηCTF is processed by β-secretase, in our conditions, we were unable to detect C99, suggesting that this fragment is either poorly generated or, alternatively, rapidly degraded or further processed into C83 as was demonstrated in other cell models [11, 17]. Taken together, our results demonstrate that ηCTF expressed in APP KO fibroblasts is indeed cleaved by α- β- and γ-secretases to yield C83, Aηα and Aβ peptides as major detectable catabolites.

### Subcellular localization of ηCTF in trans-Golgi network and in endosomes

**Fig. 4:**
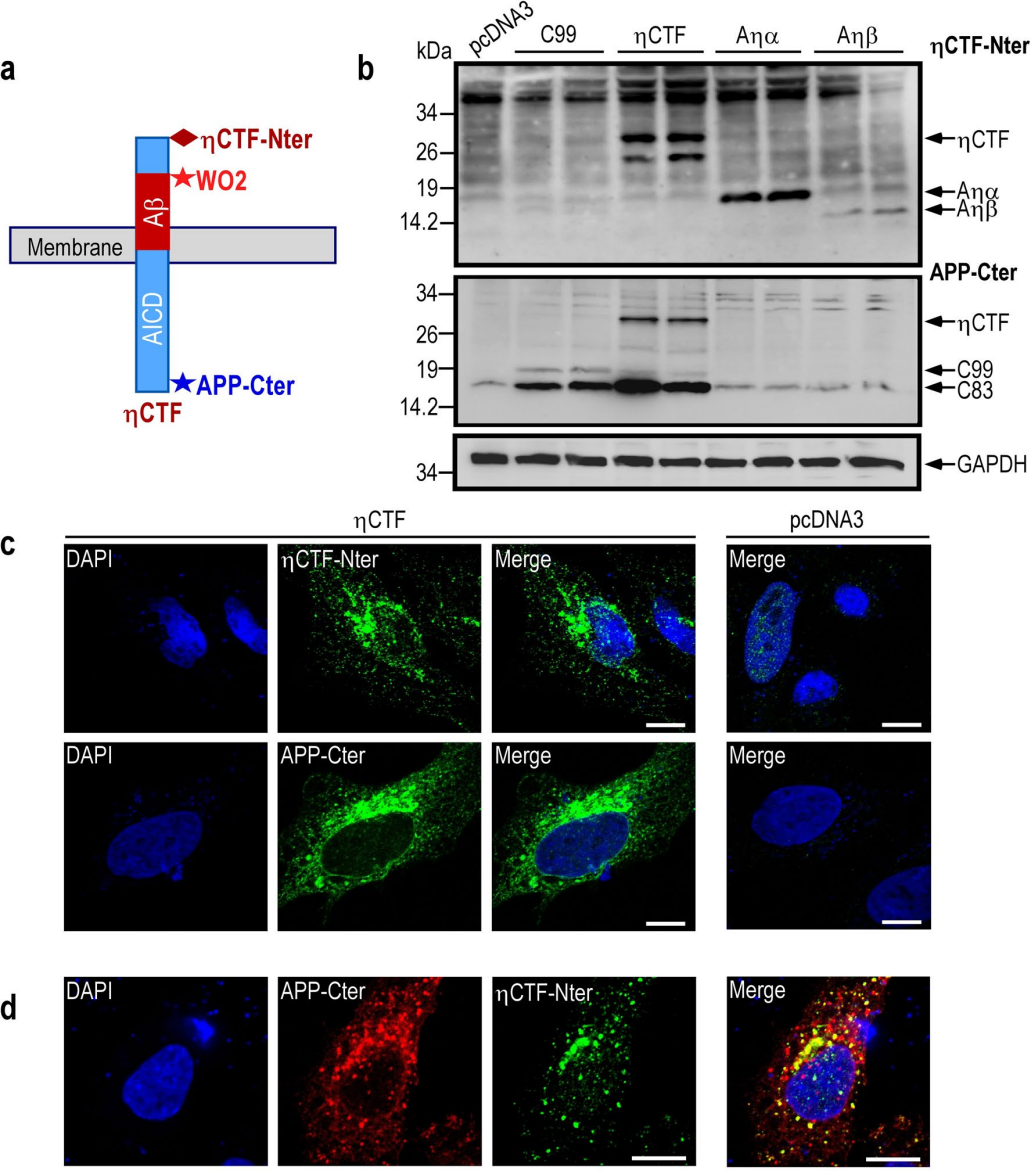
Characterization of a new η-CTF-Nter antibody. **a** Schematic illustration of antibody epitopes on ηCTF fragment. The new ηCTF-Nter antibody is directed towards the free N-terminal epitope of ηCTF. **b** SH-SY5Y cells were transiently transfected with C99, ηCTF, Aηα, Aηβ or empty pcDNA3 vectors and analyzed by western blot using ηCTF-Nter and APP-Cter antibodies. GAPDH is used as loading control. Note that as expected, ηCTF-Nter antibody recognizes ηCTF, Aηα and Aηβ but neither C99 nor C83 while APP-Cter antibody recognizes ηCTF, C99 and C83 but neither Aηα nor Aηβ. All full gels are provided in Sup Fig. 5. **c** Hela cells were transiently transfected with ηCTF or empty pcDNA3 vector and analyzed by immunofluorescence using ηCTF-Nter or APP-Cter antibodies as described in Methods. Note that both staining are mostly perinuclear with punctuate intracellular staining. **d** ηCTF-transfected Hela cells were immunostained with APP-Cter (red) and ηCTF-Nter (green) antibodies. As expected, a part of the APP-Cter staining co-localized with ηCTF-Nter staining (yellow). Nuclei were stained with DAPI. Scale bar is 10 µm. Note that in **c**, a very faint nuclear label is observed in empty pcDNA3-transfected cells that can be likely accounted for by a very low aspecific ηCTF-Nter background

Although the immunological tools available, combined to migration pattern and molecular weight analysis, allow us to firmly identify ηCTF in vitro by western blot, these antibodies do not allow a definitive identification of the fragment in situ and thus, have strong limitations for immunohistochemical analysis. In this context, we aimed at designing a new antibody referred to as ηCTF-Nter that would specifically label the N-terminus of ηCTF (Fig. 4a). By western-blot analysis, we show that this new antibody recognizes not only ηCTF, but also, as expected, the ηCTF N-terminal sequences corresponding to Aηα and Aηβ peptides (Fig. 4b). In addition, similar increase in ηCTF-Nter-like immunoreactivity was obtained in SH-SY5Y treated with proteasomal/autophagic inhibitor (Sup Fig. 2) validating the specificity of this band as well as the in vitro usefulness of ηCTF-Nter. Interestingly, C83 and C99 fragments are not recognized by ηCTF-Nter (Fig. 4b) allowing us to get rid of these CTFs and to analyze specifically the subcellular localization of ηCTF fragment in HeLa cells transiently transfected with ηCTF cDNA. The ηCTF-Nter-like immunoreactivity obtained using the new ηCTF-Nter antibody is found mainly intracellular (Fig. 4c) and as expected, this specific ηCTF staining co-localizes with a part of the APP-Cter-like immunoreactivity that likely corresponds to η-CTF, C83 and C99 (Fig. 4d). Similarly, the ηCTF-Nter labeling partly overlaps with WO2-like immunoreactivity (Sup Fig. 3).

**Fig. 5:**
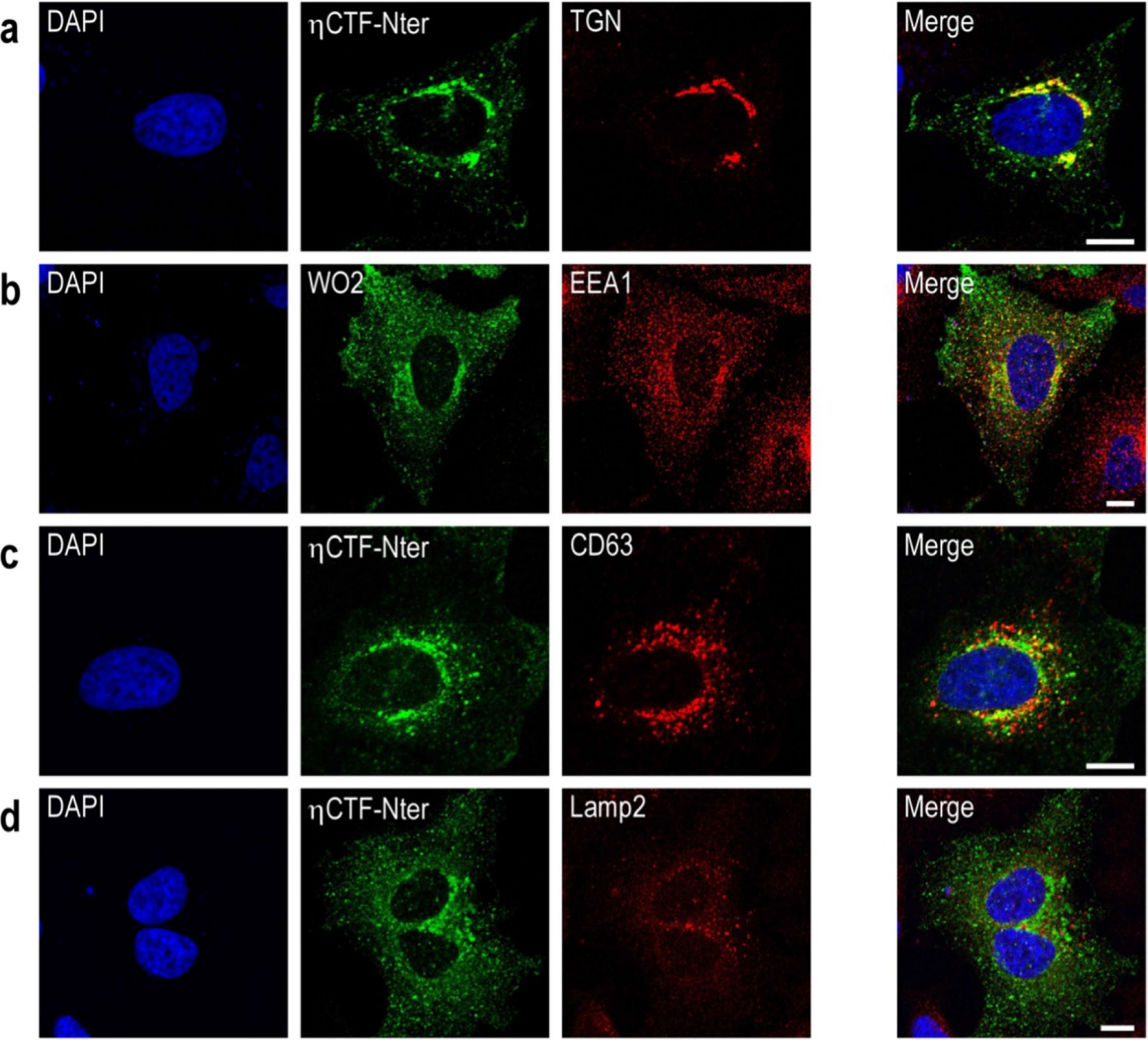
ηCTF fragment is localized in Golgi and endosomes.** a**–**d** Hela cells were transiently transfected with ηCTF and immunostained with ηCTF-Nter or WO2 (green) antibodies for ηCTF detection and antibodies directed towards TGN-46 (trans-Golgi apparatus, red, **a**), EEA1 (early endosomes, red, **b**), CD63 (late endosomes, red, **c**) or lamp2 (lysosomes, red, **d**). Note that staining corresponding to ηCTF colocalized mostly with TGN-46 and partially with EEA1 and CD63 antibodies (yellow in merge, **a**–**d**). Nuclei were stained with DAPI. Scale bar are 10 µm. Note that in **b**, WO2 was used instead of ηCTF-Nter since ηCTF-Nter and EEA1 are both rabbit antibodies and thus, preventing co-localization study

To further identify the intracellular compartments in which ηCTF-Nter-like immunoreactivity is detectable, we performed co-immunostaining using antibodies labeling different organelles. We found that ηCTF co-localizes with TGN-46, which is a marker of the Golgi apparatus and trans-Golgi network in HeLa cells (Fig. 5a). In addition, a small but significant amount of ηCTF-immunoreactivity is observed in EEA1- and CD63-positive structures corresponding to early and late endosomal compartments, respectively (Fig. 5b, c), while little if any co-staining was observed with the lysosomal marker Lamp2 (Fig. 5d).

A similar localization of ηCTF was observed in wild-type mouse organotypic hippocampal slices infected with an adeno-associated-virus (AAV) expressing ηCTF (Sup Fig. 4). We confirmed the presence of ηCTF-Nter-like immunoreactivity in EEA1-positive endosomal compartments and observed a characteristic perinuclear trans-Golgi staining and a strong overlap with APP-Cter labeling (Sup. Figure 4). It is of note that the antibody does not raise significant background even in situ.

Overall, the above set of data demonstrates the usefulness of our novel immunological probe to follow ηCTF expression in various models expressing ηCTF fragment in both in vitro (HeLa cells) and ex vivo (organotypic mouse hippocampal slices) and leads to the consistent conclusion of a main Golgi and endosomal localizations of the ηCTF fragment.

**Fig. 6:**
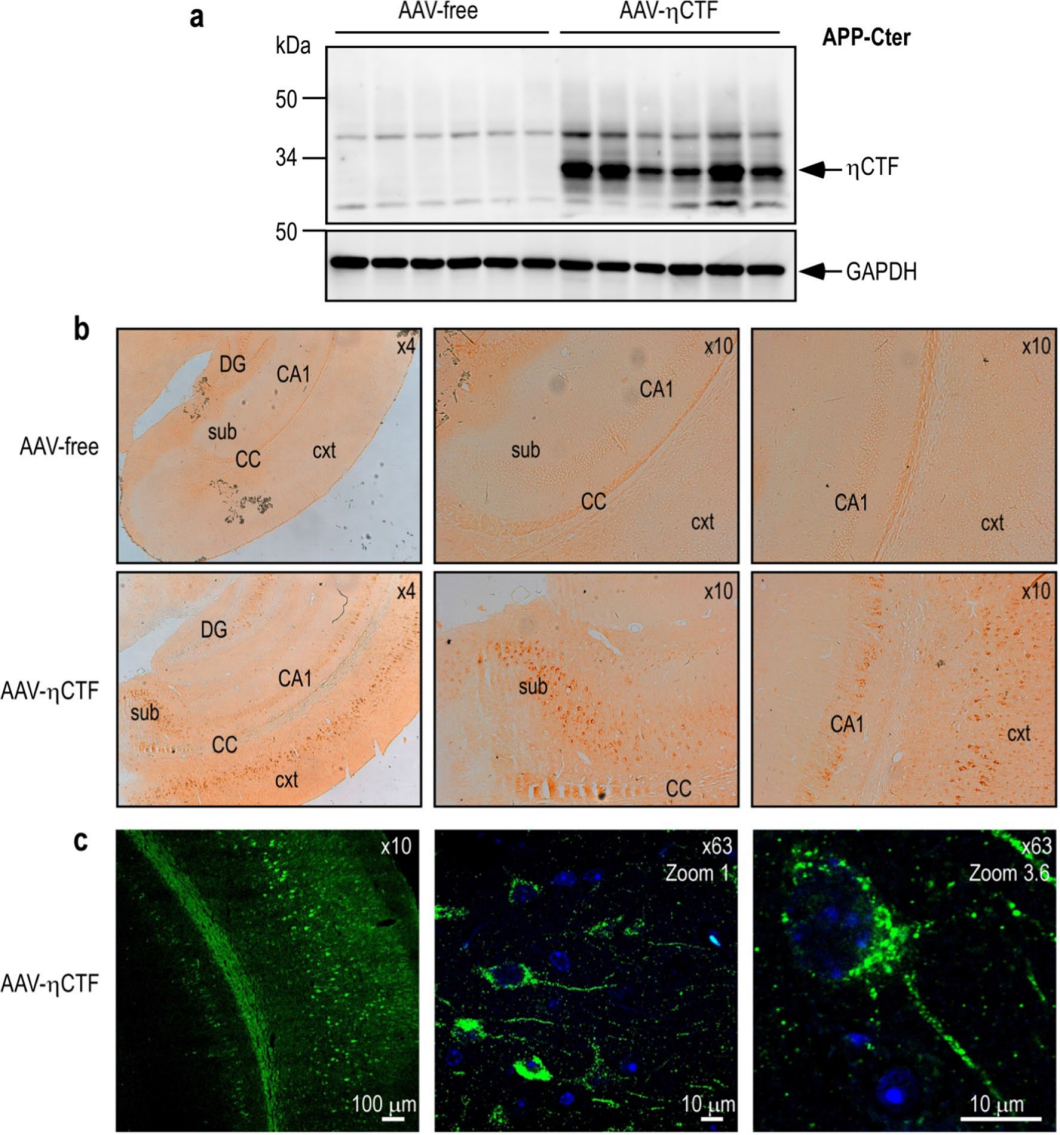
ηCTF expression and in situ localization in AAV-ηCTF mouse brains.** a**–**c** Wild-type newborn mice were infected with adeno-associated virus expressing ηCTF (AAV-η-CTF) or control empty vector (AAV-free) by intra-cerebro-ventricular (ICV) injection then sacrificed at 3-month-old. Brains were dissected and homogenized for membrane protein purification then analyzed in western blot using APP-Cter antibody. A specific band corresponding to ηCTF is detected around 30 kDa. GAPDH is used as loading control (**a**). All full gels are provided in Sup Fig. 5. Brain sections were immunostained with ηCTF-Nter antibody and revealed by horseradish peroxidase DAB (**b**) or by immunofluorescence (**c**). Brain regions are depicted as cortex (cxt), corpus callosum (CC), subiculum (sub), hippocampal CA1 region (CA1) and dentate gyrus (DG). Specific ηCTF-Nter immunostaining occurs in cortex, subiculum and hippocampus. Confocal images obtained with ηCTF-Nter antibody showed a perinuclear with punctuate intracellular staining (**c**). Nuclei were stained with DAPI

The specific ηCTF-staining was also examined in vivo, in brain slices of mice expressing ηCTF through a previously described AAV-viral strategy [13]. Western-blot analysis of ηCTF expression (Fig. 6a) and immunohistochemical assessment with η-CTF-Nter (Fig. 6b c) revealed that ηCTF is highly expressed after viral infection in brain homogenate (Fig. 6a) and is specifically detected in situ in cortex and hippocampus of AAV-ηCTF mice (Fig. 6b c).

### Detection of ηCTF fragment in small extracellular vesicles (sEVs)

**Fig. 7:**
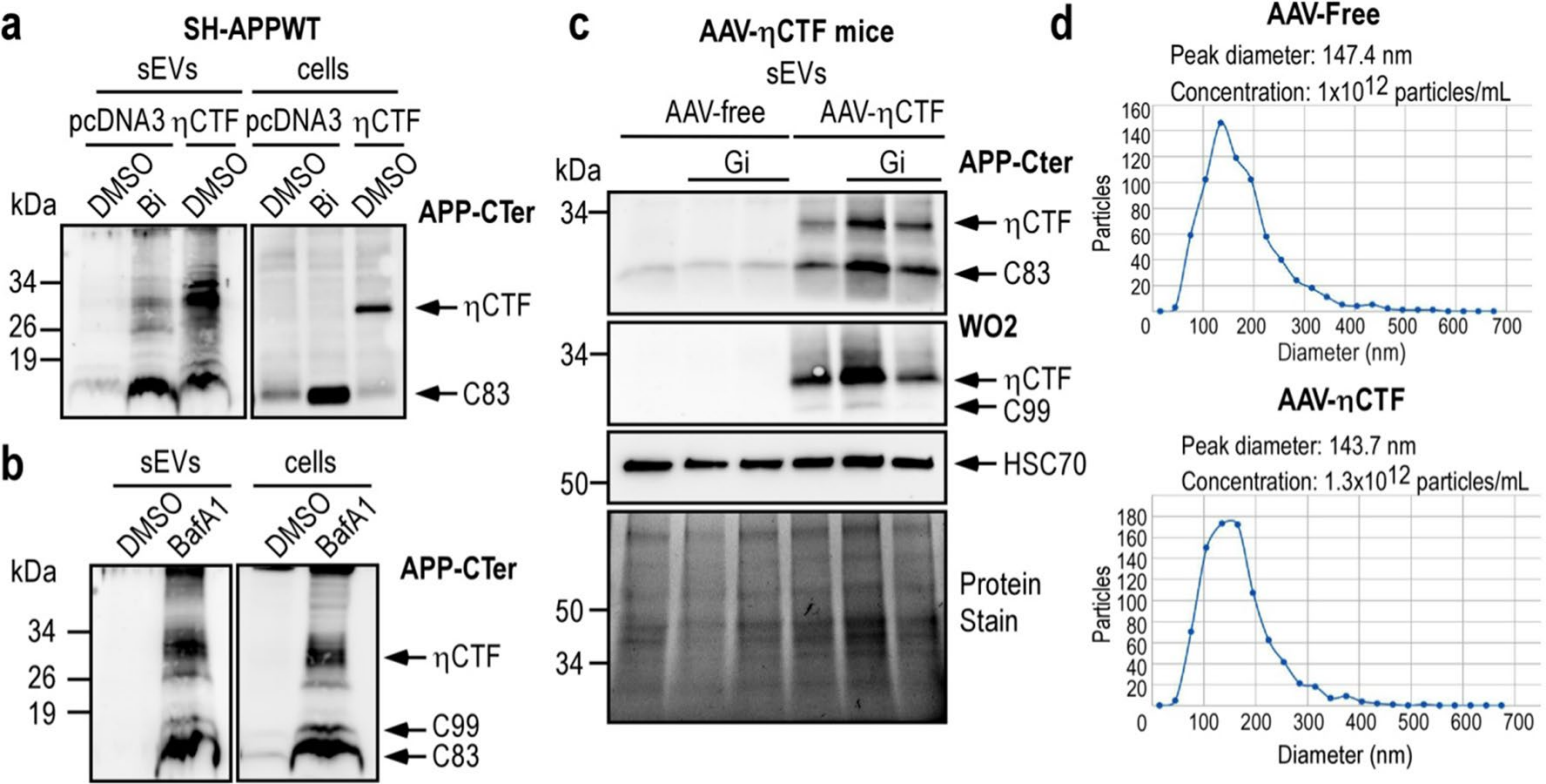
ηCTF fragment is detected in sEVs purified from cells and mouse brains. **a**–**b** SH-APPWT cells were transiently transfected with ηCTF or empty pcDNA3 vector and treated for 24 h with β-secretase inhibitor (**a**, Bi, 30 µM), or bafilomycin A1 (**b**, BafA1, 100 nM). Cell lysates and sEVs were purified from culture media as described in methods and analyzed by western blot using APP-Cter antibody. **c** sEVs were purified from brain homogenates of 3-month-old AAV-free and AAV-ηCTF mice in the presence or not of the α-secretase inhibitor (Gi:10 µM) and analyzed by western blot using APP-Cter antibody. HSC70 is used as an exosomal marker. Whole loaded proteins were stained by photoactivation using Bio-Rad prestain method (Protein Stain) as loading control. All full gels are provided in Sup Fig. 5. **d** Concentration and particles size of each brain mouse exosomal purified samples were analyzed in ZetaView instrument (Particle-Metrix) before loading on gels

The presence of the ηCTF fragment in endosomes led us to investigate whether ηCTF fragment could be found in exosomes which originate from endosomes. These small extracellular vesicles (sEVs) are currently envisioned as organelles mediating toxic spreading in various neurodegenerative diseases [22–24]. Our data show that ηCTF fragment is detectable in sEVs isolated from η-CTF-expressing cells and mice. Indeed, we detected ηCTF in sEVs purified from both media of η-CTF-expressing SH-APPWT and of β-secretase inhibitor-treated pcDNA_3_ SH-APPWT cells (Fig. 7a). As expected, we also observed high recoveries of ηCTF in sEVs following bafilomycin A1 treatment which is known to increase exosomal secretion (BafA1 Fig. 7b). Since exosomes contain high levels of α-secretases (ADAM10 and ADAM17) [25, 26] and since ηCTF was found to undergo α-secretase cleavage, sEVs were purified from mouse brains in the presence (or not) of the α-secretase inhibitor (Gi) to enhance ηCTF recovery. Our data show that ηCTF can be detected in sEVs (with a medium size around 140 nm) purified from mouse brains overexpressing ηCTF (Fig. 7c, d). This agrees well with Laulagnier and collaborators who reported the presence of ηCTF in exosomes purified from primary cortical neurons or neuroblastoma N2a cells overexpressing APP wild-type [27].

### ηCTF surrounds amyloid plaques and accumulates in an age-dependent manner in 3xTgAD mouse brains

**Fig. 8:**
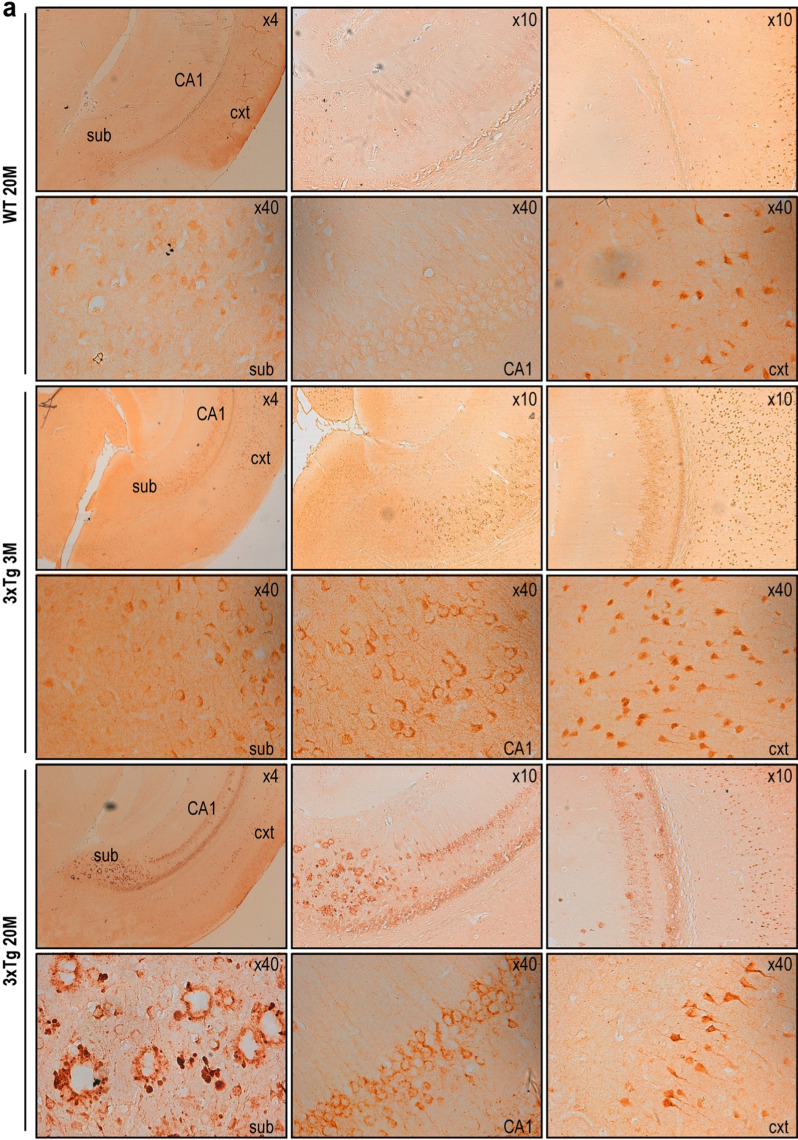

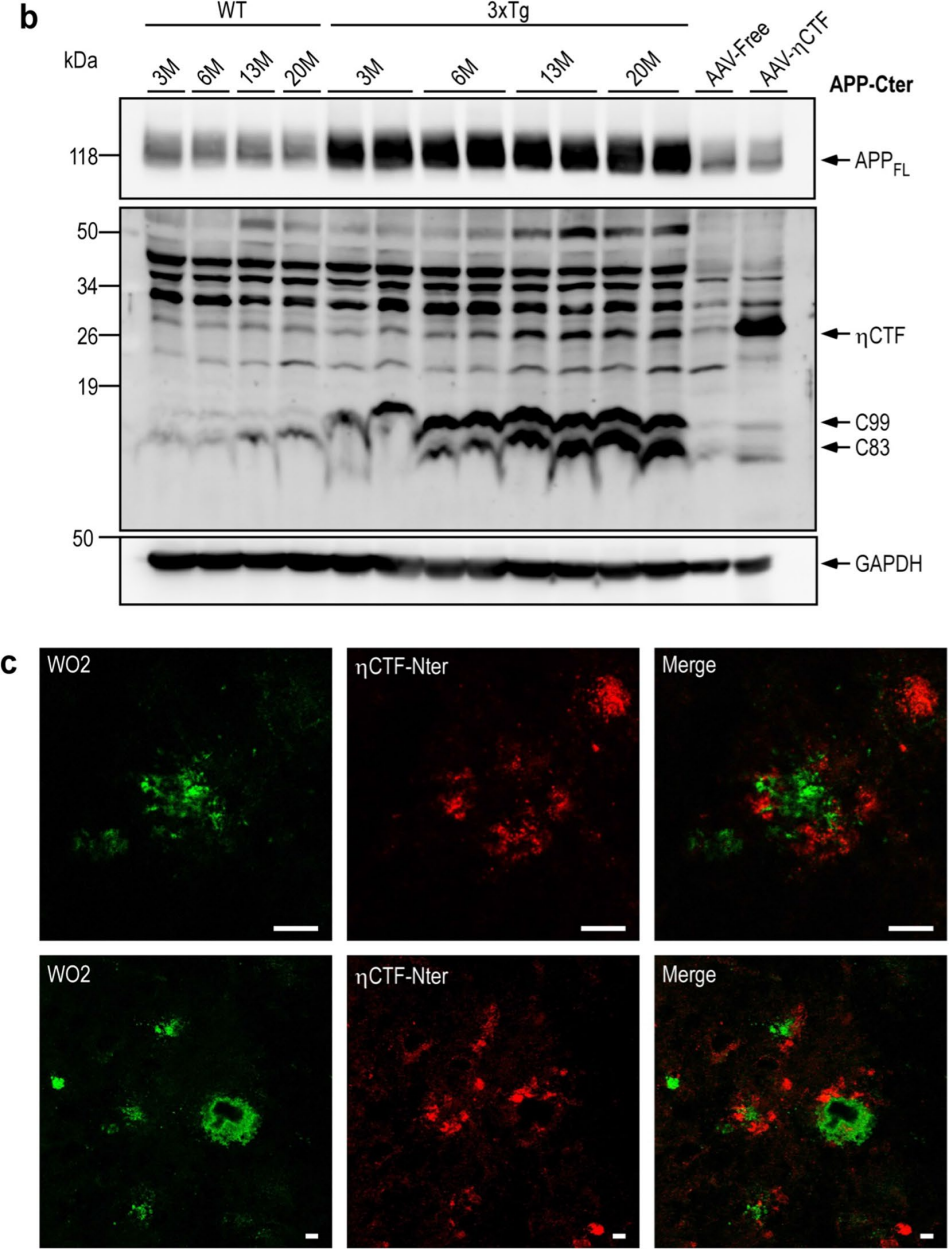
ηCTF fragment accumulates in 3xTgAD brains. **a**–**c** Brains of wild-type (WT) and triple transgenic (3xTg) females were analyzed at 3-, 6, 13- and 20-month- old by immunohistology (**a**), western blot (**b**) or immunofluorescence (**c**). DAB-immunohistochemical staining is obtained using ηCTF-Nter antibody as described in the Methods. Higher magnification reveals an intracellular labeling clearly observed in cortex, subiculum and hippocampus of 3xTg mice while a weak staining was detected only in the cortex of wild-type mice. In subiculum of 20-month-old 3xTg mice, an extracellular staining is observed around amyloid plaques (**a**). Brains of wild-type (WT), 3xTg, AAV-free and AAV-ηCTF were homogenized for membrane protein preparation then analyzed in western blot using α-APP-Cter antibody. A specific band corresponding to ηCTF is detected around 30 kDa in wild-type (WT) mice and accumulates in 3xTg mice. GAPDH is used as loading control (**b**). All full gels are provided in Sup Fig. 5. Confocal images obtained with WO2 (green) and η-CTF-Nter (red) antibodies and merged images from 20-month-old 3xTg hippocampus revealed WO2-positive core plaques surrounded by an ηCTF-Nter-like immunoreactivity (**c**)

Using the ηCTF-NTer antibody, we observed ηCTF-like immunoreactivity in the hippocampus of 3xTgAD mice, a widely used AD-mice model [14]. The labeling increased progressively from 3 to 20 months of age (Fig. 8a, lower panels) while only a very faint labeling was observed in the cortex of 20-month-old wild-type mice (Fig. 8a, upper panels). This age-related accumulation of ηCTF fragment was confirmed by western blot using the APP-Cter antibody (Fig. 8b). Interestingly, in the subiculum of 20-month-old 3xTgAD mice, the η-CTF-Nter staining obtained by peroxydase/DAB development appears more punctiform suggesting an aggregated state and is mainly surrounding plaques (Fig. 8a, lower panel). These findings were confirmed by immunofluorescence in double immunostaining experiments with ηCTF-Nter and WO2 antibodies. WO2 targeting the N-terminal part of Aβ labeled the core of the extracellular plaque while ηCTF-Nter staining surrounded WO2-associated labeling (Fig. 8c). This set of experiments confirms the usefulness of the ηCTF-Nter antibody to detect ηCTF in vivo and indicates that ηCTF accumulates at late stage of the pathology around plaques in 3xTg-AD mice.

## Discussion

The amyloid hypothesis is strongly supported by a bulk of genetic, anatomical and biochemical evidences but an increasing number of studies suggest that Aβ could likely not account for all cellular and behavioral dysfunctions taking place in AD [28]. The limited progress of Aβ-directed clinical trials suggested that other triggers could well be involved in AD etiology. In this context, to reconcile genetic grounds and clinical observations, one could envision additional APP-related fragments, distinct from canonical Aβ, as putative contributors to disease onset and/or progression. Along this line of reasoning, several tracks have been followed including numerous truncated forms of Aβ peptides [29–31] as well as the AICD transcription factor [5]. Further, our laboratory demonstrated that the β-secretase-derived APP fragment C99 could account for early dysfunctions observed in AD pathology [32, 33]. More recently, the disintegrin MT5-MMP was identified as a novel secretase named η-secretase [7, 8]. This metalloprotease triggers a cleavage of APP upstream to the one due to β-secretase, thereby yielding a fragment referred to as ηCTF, the biology of which has been poorly examined yet. In this study, we show data on the processing, fate, subcellular localization and exosomal secretion of ηCTF.

In neuronal cells, as is the case for APP, ηCTF undergoes same α- and β-secretases cleavages yielding the C-terminal fragments C83 and C99 and their N-terminal counterparts Aηα and Aηβ, respectively. Of interest, we report for the first time that ηCTF also undergoes γ-secretase-mediated cleavage giving rise to Aβ peptides. This was definitively demonstrated by our data obtained in APP KO fibroblasts where the expression of ηCTF indeed leads to Aβ40 production. In the latter cellular model, ηCTF undergoes proteolysis by α-secretase as illustrated by C83 accumulation and Aηα secretion. This α-secretase can occur at the cellular plasma membrane as previously extensively described [34] but also inside exosomes where the sheddases ADAM10 and ADAM17 (TACE) are enriched and active [25, 26]. Indeed, exosomes have been proposed to serve as a platform for ectodomain shedding of a variety of transmembrane proteins such as TNFR1, CD46, CD44 and the L1 adhesion molecule [35]. It should be noted that although β-secretase cleavage takes place on ηCTF (since Aβ is produced), C99 remained poorly detectable. This could be explained by different ways that would not be necessarily exclusive: (1) α-secretase activity is much higher than β-secretase in fibroblasts and thus, C83 is preferentially produced; (2) α-secretase displays a much higher affinity for ηCTF than β-secretase; (3) a small proportion of whole ηCTF meets active β-secretase in endosomes, (4) C99 is more rapidly degraded than C83 in fibroblasts; (5) C99 is produced but itself rapidly converted into C83 as we previously showed [11, 17]. Overall, the above set of data indicates that ηCTF behaves as a direct precursor of both C99 and Aβ peptide and thus, displays a theoretical toxic potential. This is in agreement with Baranger and colleague’s studies that proposed the η-secretase as a new pro-amyloidogenic proteinase [8, 9].

Very little is known about the fate of ηCTF and the mechanisms by which this fragment is cleared off the cells. By means of a pharmacological approach, we established that ηCTF degradation involves both proteasomal and autophagic pathways. However, the increase of endogenous C83 expression was more importantly triggered by bafilomycin A1 (Fig. 2c) than by proteasome blockers, suggesting that the autophagic process likely remains as the main degradative process of endogenous APP-CTF as previously reported [13, 19–21, 36]. The degradation of ηCTF in the autophagic pathway is in agreement with previous report by Wang and collaborators who described ηCTF fragment as a new APP-Cter fragment detected in naïves HEK293 cells following cathepsins inhibition [6]. In addition to autophagy, our result also demonstrated a significant degradation of ηCTF by the proteasome. However, it should be noted that transient or constitutive ectopic protein expression may lead to mis-localizations and/or artifactual protein aggregation. Thus, when misfolded proteins accumulate in the ER, the unfolded protein response (UPR) and ER-associated degradation (ERAD) mechanism are induced to avoid cellular damages. In line with this warning, Evrard and collaborators have shown that endogenous APP-CTFs are mainly processed by the endosomal/lysosomal pathway while overexpressed C99 was mainly degraded by the proteasome [37]. It could be the reason why in our experimental conditions, overexpressed ηCTF is readily degraded by the proteasome. In addition, our results indicate that the ηCTF overexpressed either in MEF APP KO fibroblasts or SH-APPWT neuronal cells, is not protected by the inhibition of BACE-1 activity unlike was previously described for the endogenous ηCTF [7]. This discrepancy could be also explained by the source of the ηCTF fragment (overexpressed vs endogenous).

Although molecular weight estimation and immunological characterization allows clear biochemical identification of ηCTF by western blot, this toolbox falls short when one aims at studying ηCTF in situ. In this context, we envisioned the design of a novel immunological probe that would discriminate between the plethora of APP-CTFs. Thus, we designed an antibody referred to as ηCTF-NTer that labels ηCTF N-terminus moiety and thus, would not interact with C83 and C99 or Aβ. This was successfully achieved as illustrated in Fig. 4. Thanks to this new ηCTF-Nter antibody, immunocytochemical analysis indicated that ηCTF is mostly localized in Golgi apparatus and trans-Golgi network in HeLa cells. However, a lower amount of ηCTF immunoreactivity is also observed in endosomes in both ηCTF transfected HeLa cells and hippocampal organotypic slices prepared from newborn mice expressing ηCTF.

We took advantage of our know-how in the preparation of fully characterized exosomes [17] to examine whether ηCTF could be detected in these small extracellular vesicles (sEVs). Indeed, we show the presence of ηCTF in sEVs purified from secretates of human neuroblastoma cells overexpressing ηCTF. This agrees well with the study by Laulagnier and collaborators who detected ηCTF in exosomes secreted by rat cortical neurons expressing endogenous APP protein as well as by mouse N2a cell line overexpressing wild-type APP protein [27]. Of note, we were also able to detect the ηCTF fragment derived from wild-type APP protein in sEVs purified from medium of SH-APPWT cells treated with β-secretase inhibitor. Thus, our immunohistochemical analysis of ηCTF localization and biochemical analysis of exosomal content in cells all suggest that at least a part of ηCTF is transported through endosomes and accumulate in exosomes. Exosomes can be secreted by all cell types such as neurons, oligodendrocytes, astrocytes or microglial cells and have been described to play important physiological and pathological roles in cellular communication as well as in protein aggregates spreading [38]. In AD pathogenesis, Tau protein [39], Aβ peptides [40, 41] and C99 fragment [36, 42, 43] have been found in multivesicular bodies and exosomes. Our laboratory previously established the presence of C99/C83 oligomers in exosomes and their accumulation is enhanced upon γ-secretase inhibition [17]. Thus, both C99 and its precursor ηCTF accumulate similarly in exosomes.

Finally, we also documented the usefulness of our novel immunological probe for in vivo approaches and demonstrated the presence of ηCTF in exosomes purified from brains of mice expressing ηCTF. Moreover, we were able to show a high ηCTF expression surrounding the core of abundant senile plaques in triple transgenic mice as was also shown in APP/PS1 transgenic mice and in AD brain [7].

A question arises as to whether ηCTF could be considered as a fully toxic trigger in AD or could govern more balanced physiopathological functions. In this regard, similarly to Aβ peptides, C99 or phosphorylated Tau proteins, the presence of ηCTF fragments in exosomes could have both beneficial or deleterious consequences. Exosomes carrying ηCTF could contribute (1) to eliminate excess of ηCTF from the cells; (2) to transport physiological intercellular signals or (3) to be responsible for neuropathological spreading [38]. Nonetheless, the presence of ηCTF in exosomes as well as its accumulation and detection around amyloid plaques may support its potential contribution to AD pathology. This postulate is strengthened by the fact that ηCTF acts as a direct precursor of C99 that is considered as an early trigger of AD pathology [44]. Therefore ηCTF could be a source of intracellular C99/Aβ production but also an indirect way to promote the exosomal C99/Aβ spreading resulting in an extracellular Aβ deposits. In addition, exosomes released from neurons have been described to regulate synaptic activity [45]. Further, ηCTF yields Aηα and Aηβ, the influence of which on hippocampal long-term potentiation has led to discrepant conclusions [7, 46]. Apparently, Aηα and Aηβ peptides could impair synaptic activity [46]. This adds support to the conclusion that ηCTF directly per se, or indirectly, as a precursor, could well contribute to AD pathology. Thus preventing ηCTF production could be of therapeutic relevance to act at very early stages of AD. This newcomer on disease stage could reconcile, at least to some extent, the numerous failures of clinical trials targeting Aβ with the well supported genetic hypothesis centered on APP.

### Supplementary Information

Below is the link to the electronic supplementary material.

Supplementary file1 (DOCX 2200 KB)

## Data Availability

Not applicable. All full gels are provided in Supplementary Fig. 5.
