# Supplementary material for: The η-secretase-derived APP fragment ηCTF is localized in Golgi, endosomes and extracellular vesicles and contributes to Aβ production
Source: Cell Mol Life Sci. 2023 Mar 17;80(4):97. doi: 10.1007/s00018-023-04737-4 (PMC10023608; doi:10.1007/s00018-023-04737-4)

**Supplementary Materials and Methods**

**Organotypic hippocampal slices preparation, culture and immunostaining**

Organotypic hippocampal slices were obtained from postnatal day 5-7 C57BL6JRj mice as previously described (1). Briefly, hippocampi were sliced into 400 µm sections and carefully transferred on sterile hydrophilic membrane millicell discs (Millipore, FHLC01300) placed in semiporous cell culture inserts (Millipore, 0.4 µm) within a 6-well plate, balanced with the warm culture medium (Minimum Essential Medium Eagle (MEM)+Glutamax-1 (50%), EBSS (18%), EBSS (13%)/D-glucose (5%), penicillin–streptomycin (5000U/ml, 1%), Horse serum (25%) and Nystatin 10000U/ml (0.06%). Slices were infected 2 h after plating with adeno-associated viruses AAV-free or AAV-ηCTF: 5 x 10^12^ vg/ml). Slices were incubated at 37°C, 5% CO2 and half of the medium was replaced every 3 days. After 9 days in culture, slices were fixed for 20 min in paraformaldehyde 4% then stored at 4°C in PBS and kept for immunostaining.

For immunostaining, organotypic hippocampal slices were permeabilized overnight in PBS Triton (0.4%), blocked with BSA (5%)/Tween20 (0.1%), then incubated with primary antibodies: α-APPct, mouse monoclonal (1:1000, Biolegend) or ηCTF-Nter (1:800) for ηCTF detection or/and α-EEA1 goat polyclonal (1:1000) for early endosomes labelling. After washes, coverslips were incubated with Alexa Fluor-488, Alexa Fluor-590 and Alexa Fluor-647 conjugated antibodies (1:1000) and DAPI (1: 20,000) staining. Finally, the sections were washed with PBS, then mounted onto glass slides and cover-slipped. The stained slices were kept at 4◦C before analysis with confocal microscopy (Zeiss LSM 780 with 63X Objective).

**Supplementary legends to figures**

**Sup Fig 1: Autophagy and β-secretase inhibitions enhance ηCTF level in SH-APPWT cells.**

**a-c.** SH-APPWT cells were transiently transfected with ηCTF-pcDNA_3_ or empty pcDNA_3_ vector and treated for 24h with bafilomycin A1 (BafA1, 100nM) or Smer28 (50µM) or with α- β- or γ-secretase inhibitors (Gi:10µM, Bi:30µM, D6:1µM), then analyzed by western-blot using APP-Cter, WO2 or 82E1 antibodies where indicated. GAPDH is used as loading control. Histograms in **c** correspond to the quantification of ηCTF immunoreactivity obtained in **b** in ηCTF-expressing SH-APPWT. Values are expressed as percentage of DMSO-treated cells (control) taken as 100, and are the means +/- S.E.M of 11 independent determinations. ns: not statistically significant according to the Tukey one-way ANOVA test. When probed with WO2 and 82E1, the same blots are revealed following short and long exposure (exp). Note that in mock SH-APPWT cells, a band migrating around 30kDa was detected with APP-Cter and WO2 antibodies following bafilomycin A1 treatment (**a**) and β-secretase inhibition (**b**) following long exposure. All full gels are provided in Sup Fig 5.

**Sup Fig. 2: ηCTF-Nter-like immunoreactivity is recovered upon both proteasome and autophagy inhibitions.**

**a-b.** SH-SY5Y cells were transiently transfected with ηCTF or pcDNA3 vector and treated for 24h with proteasome inhibitors (**a**, lactacystine (Lact, 5µM), epoxomicin (Epox, 1µM), MG132 (5µM)), with a lysosomal inhibitor (E-64D) or with bafilomycin A1 (BafA1, 100nM) or Smer28 (50µM) that blocks or activates autophagy respectively (**b**) then analyzed by western-blot using ηCTF-Nter antibody.

**Sup Fig 3: ηCTF Staining obtained with ηCTF-Nter antibody colocalized with WO2 staining.**

**a-b.** Hela cells were transiently transfected with ηCTF-pcDNA3 and analyzed by immunofluorescence using WO2 alone (**a**) or in combination with η-CTF-Nter (**b**) as described in the Methods. Merges (**a-b**, right panels) show no overlap between WO2 and DAPI (**a**) while, as expected, part of the WO2 staining co-localized with η-CTF-Nter staining (**b**). Nuclei were stained with DAPI.

**Sup Fig 4: η-CTF fragment is localized in early-endosomes in organotypic hippocampal slices.**

**a-b.** Organotypic hippocampal slices prepared from wild-type mice as described in supplementary Methods, were infected with AAV-free or AAV-ηCTF viruses (5 x 10^12^ vg/ml) and analyzed by immunofluorescence using ηCTF-Nter (green) in combination with APP-Cter (red in **a**) or α-EEA1 (red in **b**) antibodies as an early endosomal compartment marker. Merge images indicate that ηCTF-Nter staining colocalized with APP-Cter staining (**a,** right panel) as well as with EEA1 staining (**b,** right panel).

**Sup Fig 5: Full gels of each western-blot from all figures are provided**

**Supplementary Reference**

1. Valverde, A., Dunys, J., Lorivel, T., Debayle, D., Gay, A. S., Caillava, C., Chami, M., and Checler, F. (2021) Dipeptidyl peptidase 4 contributes to Alzheimer's disease-like defects in a mouse model and is increased in sporadic Alzheimer's disease brains. *J Biol Chem* **297**, 100963


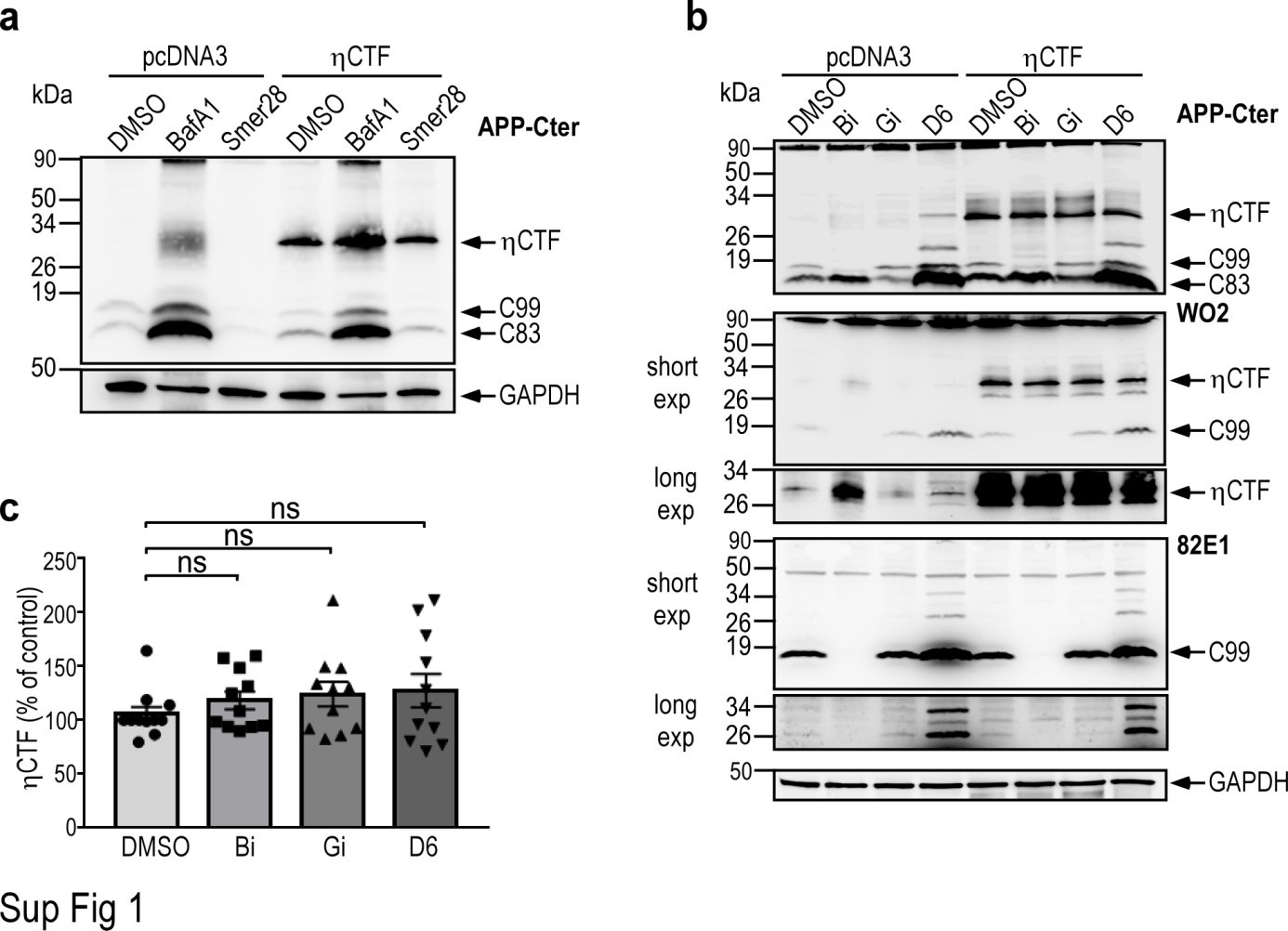


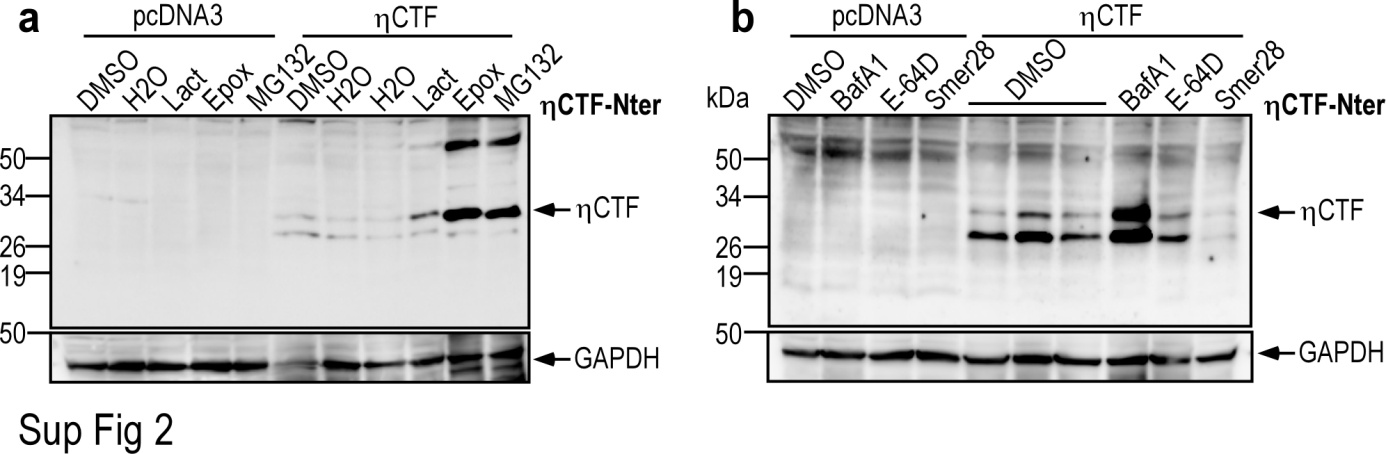


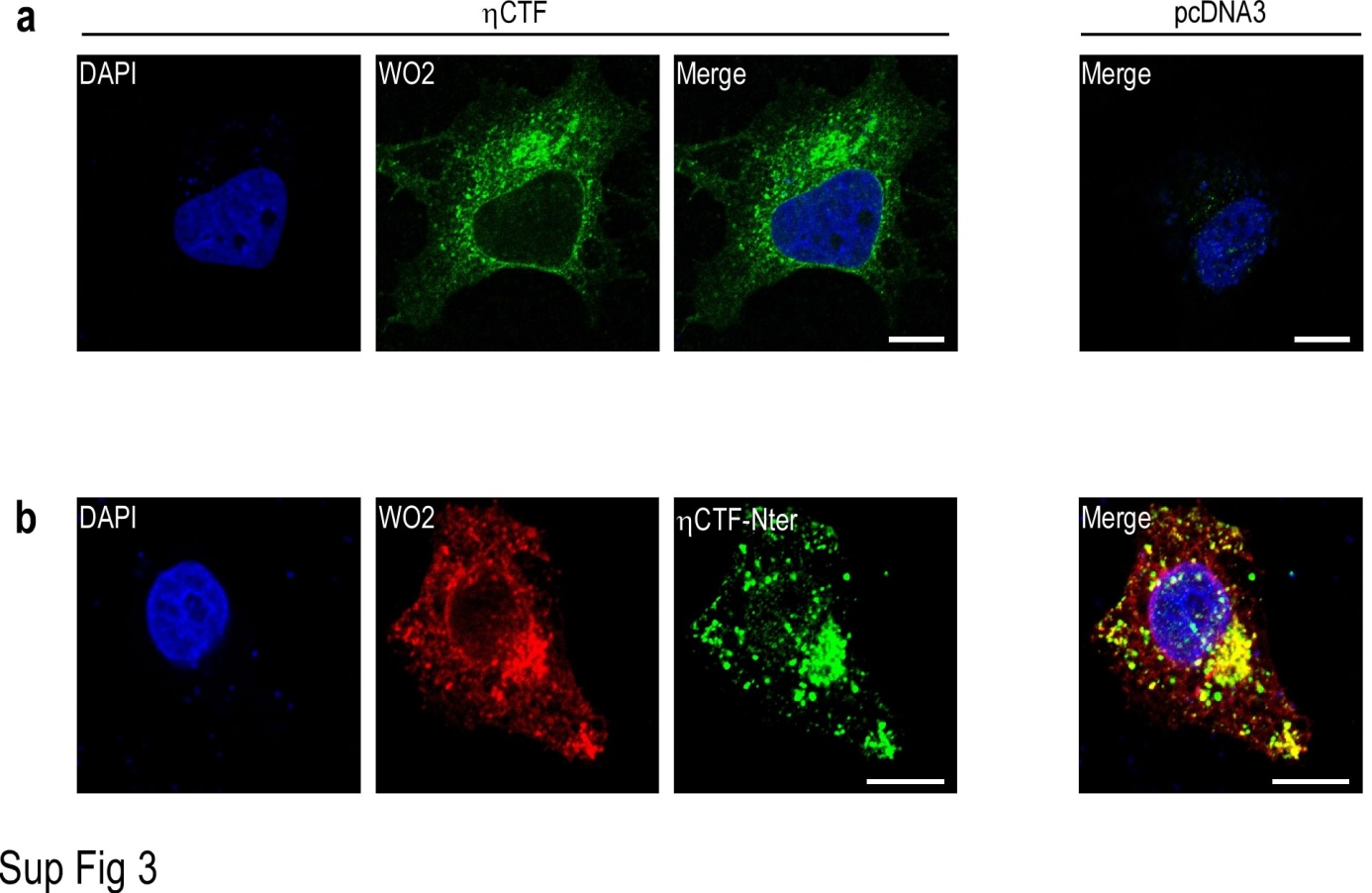


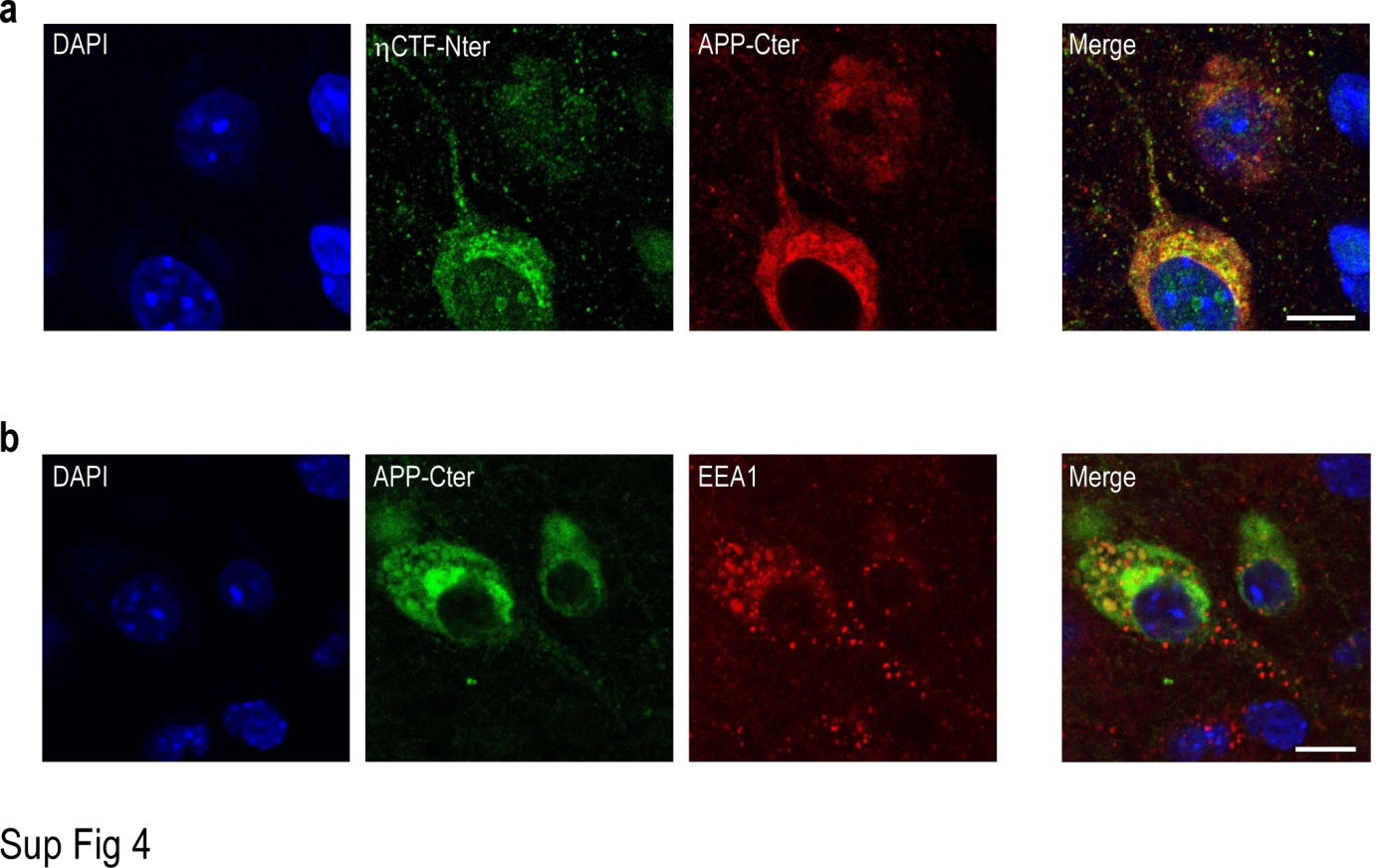


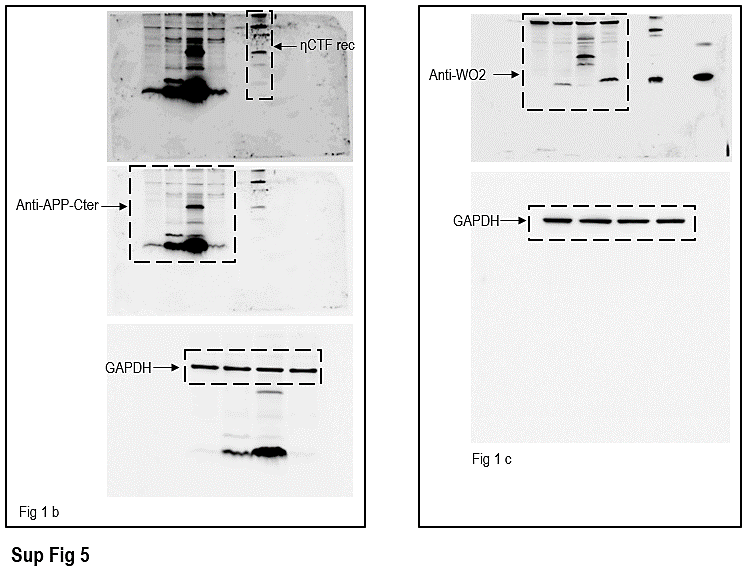


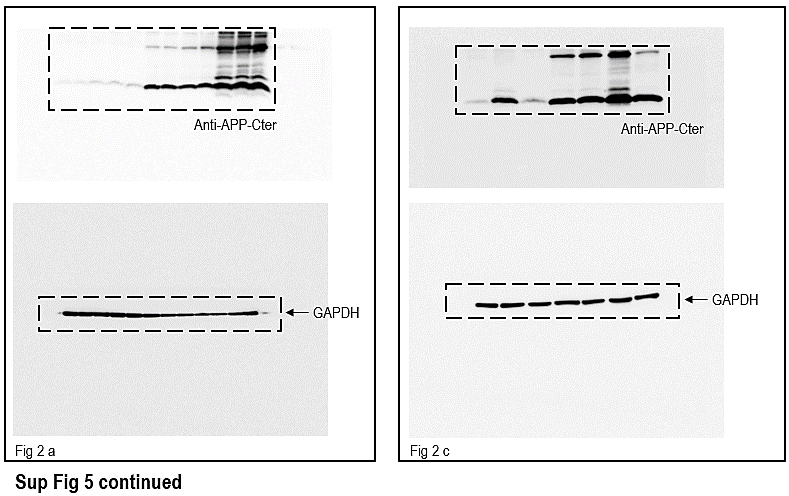


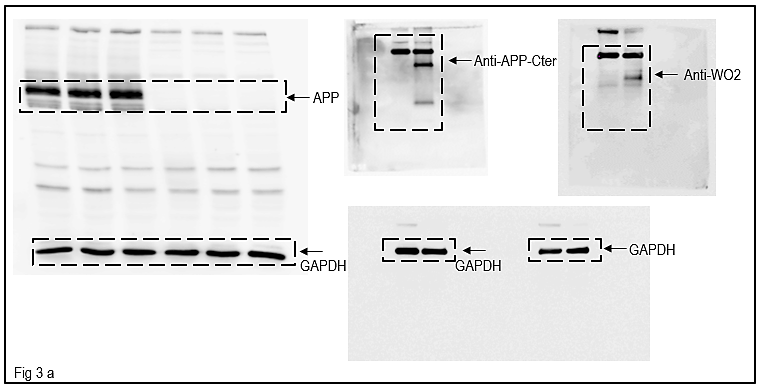


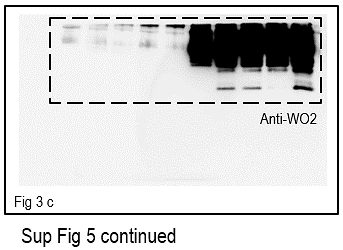


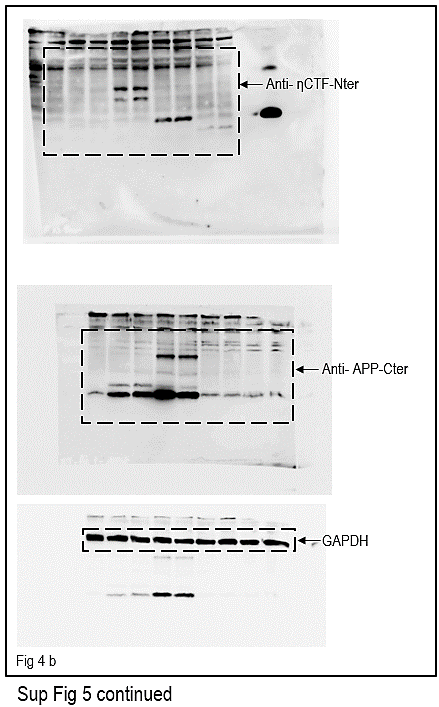


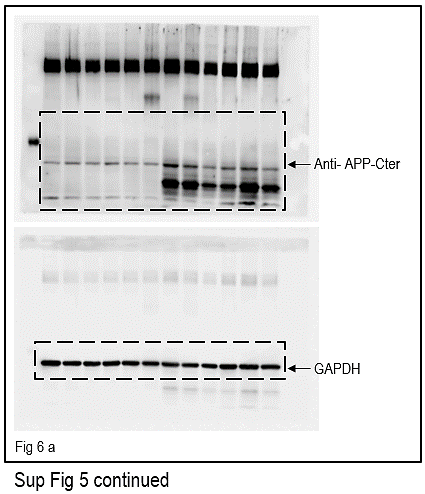


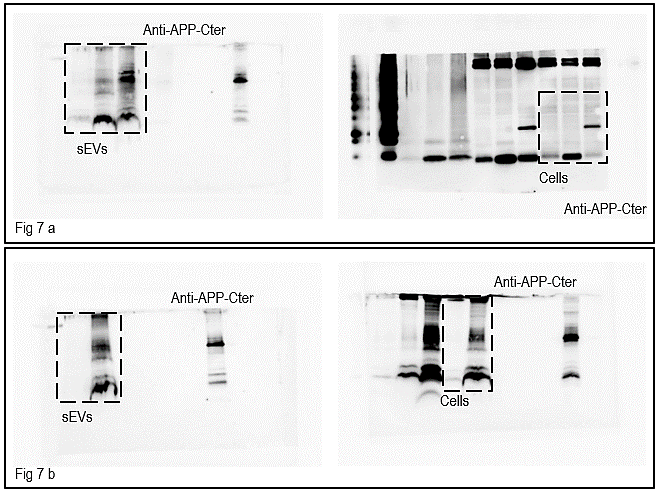


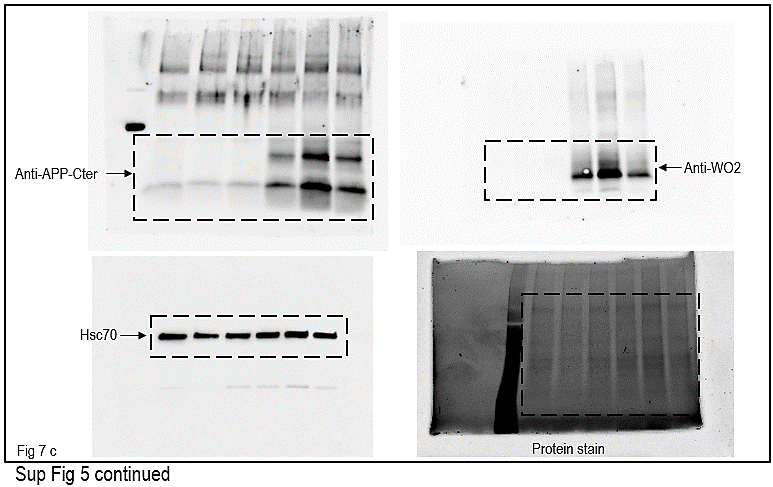


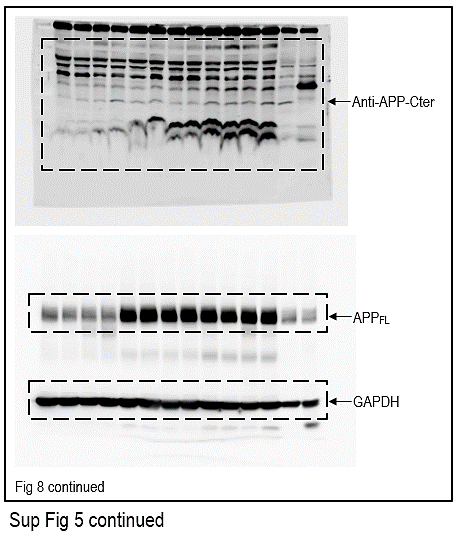


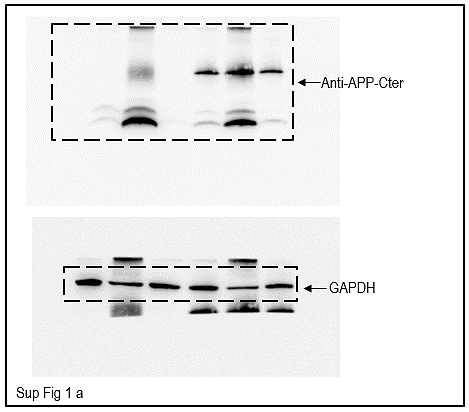


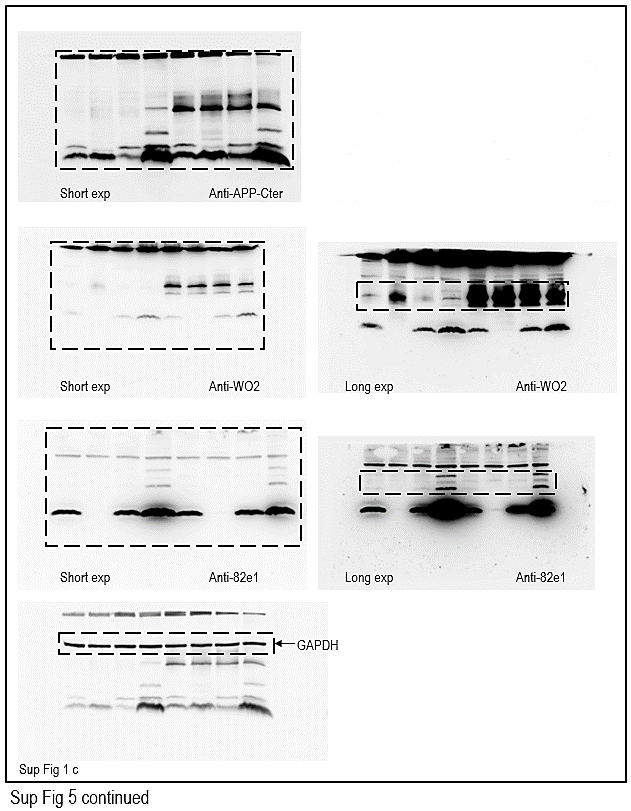


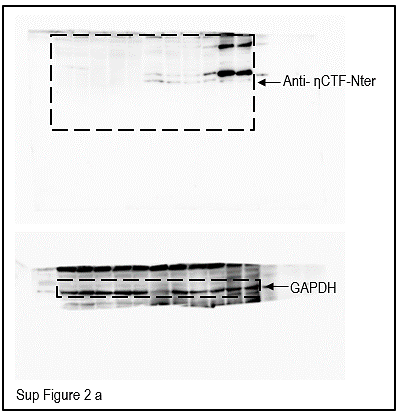


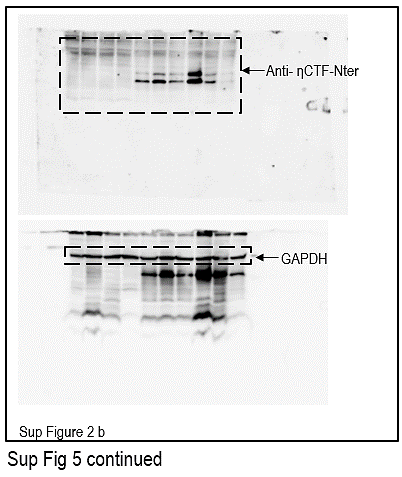

Supplement: Supplementary file 1 — Supplementary file1 (DOCX 2200 KB) [file 18_2023_4737_MOESM1_ESM.docx]
